# Functions of Nonmuscle Myosin II in Assembly of the Cellular Contractile System

**DOI:** 10.1371/journal.pone.0040814

**Published:** 2012-07-13

**Authors:** Maria Shutova, Changsong Yang, Jury M. Vasiliev, Tatyana Svitkina

**Affiliations:** 1 Department of Biology, University of Pennsylvania, Philadelphia, Pennsylvania, United States of America; 2 Institute of Carcinogenesis, Cancer Research Center, Russian Academy of Medical Sciences, Moscow, Russia; King's College London, United Kingdom

## Abstract

The contractile system of nonmuscle cells consists of interconnected actomyosin networks and bundles anchored to focal adhesions. The initiation of the contractile system assembly is poorly understood structurally and mechanistically, whereas system’s maturation heavily depends on nonmuscle myosin II (NMII). Using platinum replica electron microscopy in combination with fluorescence microscopy, we characterized the structural mechanisms of the contractile system assembly and roles of NMII at early stages of this process. We show that inhibition of NMII by a specific inhibitor, blebbistatin, in addition to known effects, such as disassembly of stress fibers and mature focal adhesions, also causes transformation of lamellipodia into unattached ruffles, loss of immature focal complexes, loss of cytoskeleton-associated NMII filaments and peripheral accumulation of activated, but unpolymerized NMII. After blebbistatin washout, assembly of the contractile system begins with quick and coordinated recovery of lamellipodia and focal complexes that occurs before reappearance of NMII bipolar filaments. The initial formation of focal complexes and subsequent assembly of NMII filaments preferentially occurred in association with filopodial bundles and concave actin bundles formed by filopodial roots at the lamellipodial base. Over time, accumulating NMII filaments help to transform the precursor structures, focal complexes and associated thin bundles, into stress fibers and mature focal adhesions. However, semi-sarcomeric organization of stress fibers develops at much slower rate. Together, our data suggest that activation of NMII motor activity by light chain phosphorylation occurs at the cell edge and is uncoupled from NMII assembly into bipolar filaments. We propose that activated, but unpolymerized NMII initiates focal complexes, thus providing traction for lamellipodial protrusion. Subsequently, the mechanical resistance of focal complexes activates a load-dependent mechanism of NMII polymerization in association with attached bundles, leading to assembly of stress fibers and maturation of focal adhesions.

## Introduction

Myosin II-dependent contraction is a universal cellular mechanism to generate pulling force through mutual sliding of actin and myosin II filaments. The cellular contractile machinery has highly ordered and stable sarcomeric organization in striated muscle [1], but is more dynamic and less orderly organized in nonmuscle cells [2], [3], [4]. The nonmuscle contractile system plays important roles in cell motility, cytokinesis, cell shape determination, cell-matrix and cell-cell junction formation and also serves as a template for assembly of sarcomeric organization in striated muscles [5].

Myosin II, a central player during cell contraction, is an actin-dependent molecular motor moving toward the plus (barbed) end of the actin filament. The hexameric myosin II molecule consists of two heavy chains containing a motor domain and dimerizing through coiled-coil tails and two pairs of light chains, essential and regulatory. Myosin II is the only member of the myosin superfamily that can assemble into bipolar filaments with motor domains positioned at both ends of the filament. When presented to actin filaments of opposite polarity, bipolar filaments cause contraction. Assembly into bipolar filaments is considered necessary for myosin II functions. Because of the presence of multiple actin-binding sites, myosin II filaments also function as cross-linkers. As compared to very long (∼ 2 µm) bipolar filaments formed by skeletal muscle myosin II, nonmuscle myosin II (NMII) forms similar, but shorter (∼ 0.3 µm) bipolar filaments. NMII is regulated primarily by phosphorylation of two conserved residues, Ser19 and Thr18, in the myosin regulatory light chain (MRLC) [6]. In the nonphosphorylated state, NMII molecules acquire a folded 10S conformation, in which they lack the ATPase, actin-binding, and polymerization activities. Phosphorylation of Ser19 unfolds the NMII molecule into extended 6S conformation and is sufficient for restoration of all these activities, but additional phosphorylation of Thr18 enhances activation [6], [7]. The bipolar filament assembly, but not motor activity, of NMII is also regulated at the NMII heavy chain level by inhibitory phosphorylation or binding of regulatory proteins, such as S100A4/Mts1 [8].

Migration of nonmuscle cells is a cycle of protrusion, adhesion, and contraction of the cell. While leading edge protrusion is driven by polymerization of actin filaments [9], contractile forces generated by NMII filaments are most evidently involved in the retraction of the cell rear and maturation of adhesion sites [2], [3], [4]. The contractile system of a nonmuscle cell consists of interconnected actin-NMII networks and bundles associated with specialized adhesion sites, such as focal adhesions [4]. Focal adhesions are initiated within lamellipodia as very dynamic nascent adhesions [10], become partly stabilized forming dot-like focal complexes at the lamellipodial base [11], and then grow, elongate, and become further stabilized producing elongated mature focal adhesions. Maturation is a force-dependent process [12] requiring the motor activity of NMII [13] or external force [14]. In contrast, nascent adhesions are formed under lamellipodia in an NMII-independent manner, whereas their transition to focal complexes requires only the cross-linking, but not motor activity of NMII [10], as well as retrograde flow of the overlaying lamellipodial network [15], [16].

In contrast to relatively well investigated pathways of initiation and maturation of focal adhesions, the structure and remodeling of newly formed actin-NMII assemblies are poorly understood. The nascent stress fibers were proposed to form in lamella from actin-NMII clusters [17], [18], or using filopodial bundles as seeds [19], [20], or through intermediate formation of transverse arc-shaped bundles formed by endwise association of two distinct sets of short actin bundles [21], [22]. However, because these models are commonly derived from light microscopic analyses that do not resolve individual filaments, the structural relationship between forming adhesions and nascent actin-NMII arrays in these non-exclusive scenarios remain uncharacterized at high resolution.

We aimed to address the above question by applying platinum replica electron microscopy (EM) to nonmuscle cells acutely reforming their contractile system after its complete disassembly. Inhibition of NMII that causes disassembly of stress fibers and focal adhesions [2], [3], [4] appeared as a suitable way to achieve this goal. Among available inhibitory approaches, only drug treatment, but not RNAi or gene knockout, is compatible with the acute recovery experiments. Many of commonly used drugs inhibit NMII by targeting an individual MRLC kinase, but may leave other MRLC kinases active. Therefore, a direct NMII inhibitor blebbistatin [23], is the best choice for this purpose. Blebbistatin inhibits the NMII motor activity by slowing down the phosphate release after ATP hydrolysis, thus blocking NMII in a weak actin-binding state [24]. Among tested myosins, blebbistatin strongly inhibits NMII and striated muscle myosin II isoforms, but does not inhibit several unconventional myosins and has a much weaker and species-specific activity against smooth muscle myosin II [23]. Therefore, in nonmuscle cells NMII is currently considered to be the major target of blebbistatin.

In this study, we completely disassembled the contractile system in spreading REF52 cells by blebbistatin treatment and then induced its acute recovery by washing out the drug. Using platinum replica EM correlated with light microscopic visualization of focal adhesions and focal complexes in the same cells, we characterized the cytoskeletal organization of the contractile system during early stages of the contractile system formation. Unexpectedly, in the course of this study we have found that blebbistatin treatment also inhibits attachment and productive protrusion of lamellipodia, whereas recovery of these structures appears to depend on activity of non-polymerized NMII molecules.

## Results

**Figure 1:**
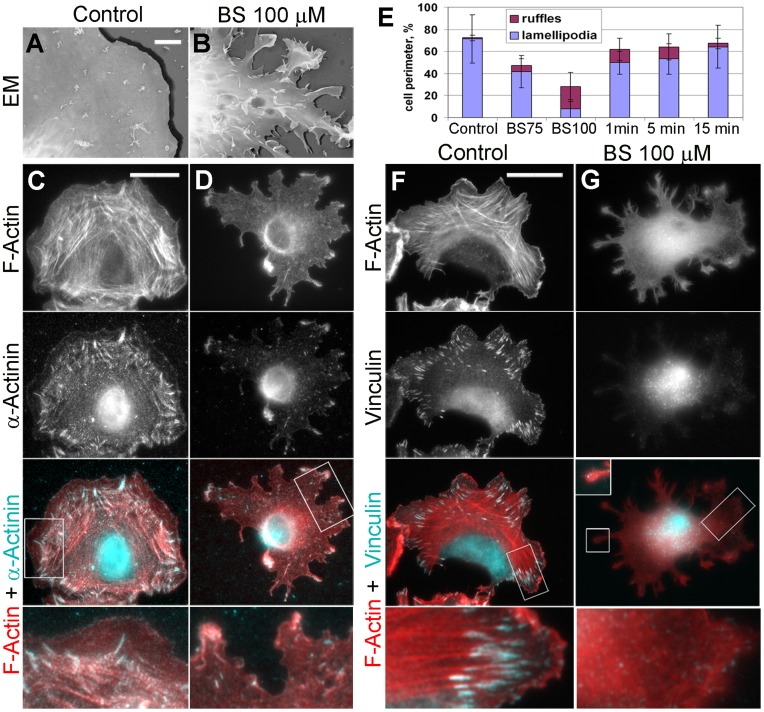
Blebbistatin inhibits lamellipodia and focal complexes in REF52 fibroblasts. (A,B) Cell surface topography revealed by platinum replica EM of non-extracted untreated (A) or 100 µM blebbistatin (BS)-treated cells (B). (C,D) Fluorescence microscopy of phalloidin-stained F-actin and immunostained α-actinin in detergent-extracted untreated (C) or 100 µM blebbistatin-treated cells (D). (E) Fractions of the cell perimeter occupied by lamellipodia and ruffles in untreated cells, cells treated with 75 µM or 100 µM blebbistatin, and cells recovering from treatment with 100 µM blebbistatin for indicated periods of time in minutes. Error bars, SD. (F,G) Fluorescence microscopy of phalloidin-stained F-actin and immunostained vinculin in detergent-extracted untreated (F) or 100 µM blebbistatin-treated (G) cells. Boxed regions in C, D, E, and G are zoomed in bottom panels. Scale bars, 2 µm (A,B) and 20 µm (C,D,F,G).

We used REF52 fibroblasts, because we have previously characterized their NMII-containing contractile apparatus by light and EM [18], [25]. In preliminary experiments, we tested a range of blebbistatin concentrations to determine a minimal concentration causing complete disassembly of stress fibers. Blebbistatin was originally reported to inhibit cytokinesis as a 100 µM mixture of active (−) and inactive (+) enantiomers [23]. Subsequent studies predominantly used 100 µM pure (−)-blebbistatin, as it allows to achieve more complete NMII inhibition without adverse effects [19], [26], [27], [28], [29], [30], [31], [32], [33], [34]. We also found that whereas a subset of stress fibers persisted in the cell interior after treatment with 50 µM [35] or 75 µM blebbistatin (Figure S1), 100 µM blebbistatin virtually completely eliminated stress fibers in spreading REF52 cells (Figure 1). Therefore, here we primarily used 100 µM (−)-blebbistatin, but also made some comparisons to the effects of 75 µM blebbistatin. To control for specificity of blebbistatin effects, we used equivalent concentrations of inactive (+)-blebbistatin and DMSO in parallel samples. In all cases, cells treated with control compounds were indistinguishable from untreated cells indicating that the observed effects were specific (Figure S2A). In subsequent sections, we first characterize the effects of blebbistatin treatment, and then how cells recover after blebbistatin washout.

### NMII Functions are Required for the Formation of Lamellipodia and Focal Complexes

Examination of the cell surface topography by platinum replica EM of nonextracted cells (Figure 1A) revealed that control cells had flat attached lamellipodia at the cell periphery. However, many ruffles appeared at cell edges after treatment with 75 µM blebbistatin (Figure S1A), whereas treatment with 100 µM blebbistatin caused severe cell retraction and formation of multiple ruffles and filopodia on the entire dorsal surface (Figure 1B). These results suggest that NMII inhibition compromises adhesion of lamellipodia. Consistent with these results, phalloidin staining showed that flat lamellipodia in control cells occupied most of the cell perimeter, while ruffles were rare (Figure 1C, E, and F). Treatment with 100 µM blebbistatin drastically shifted this balance from lamellipodia to ruffles and caused significant cell retraction (Figure 1D, E, and G), whereas 75 µM blebbistatin had similar, but less prominent effects (Figure S1B-E). As expected, blebbistatin also caused disassembly of stress fibers, which was incomplete in cells treated with 75 µM blebbistatin (Figure S1), but very extensive after treatment with 100 µM blebbistatin (Figure 1D and G). Inhibition of lamellipodia by the drug was especially clearly seen after immunostaining of α-actinin (Figure 1C and D). In control cells, α-actinin was abundant in focal adhesions, lamellipodia, and stress fibers, where it formed a striated pattern, as in other nonmuscle cells [36]. After treatment with 75 µM blebbistatin, α-actinin staining in lamellipodia remained very distinct (Figure S1B), but it virtually disappeared after treatment with 100 µM blebbistatin except for a few ruffles at the tips of extended processes (Figure 1D). Staining of α-actinin in stress fibers and focal adhesions was greatly diminished at both blebbistatin concentrations.

Distribution of focal adhesions in control and blebbistatin-treated cells was evaluated by immunostaining of vinculin (Figure 1F and G), a focal adhesion marker [37]. Control cells contained mature elongated focal adhesions at the tips of stress fibers and dot-like focal complexes located primarily at the cell periphery (Figure 1F). After treatment with 75 µM blebbistatin, mature focal adhesions disappeared, but focal complexes remained abundant, especially at the base of lamellipodia (Figure S1C), as reported in other studies [3], [10], [35], [38], [39], [40], [41]. However, treatment with 100 µM blebbistatin dramatically decreased the abundance and brightness of focal complexes (Figure 1G). The remaining weak vinculin puncta could be detected at the tips of adherent processes only after removal of the cytosolic pool of vinculin by detergent extraction. The dramatic loss of focal adhesions and focal complexes after treatment with 100 µM blebbistatin led to very poor cell-to-substrate adherence, as evidenced by massive detachment of cells during culture and handling. Thus, deep inhibition of NMII by 100 µM blebbistatin inhibited focal complexes and lamellipodia, whereas a low level of NMII activity presumably remaining after treatment with 75 µM blebbistatin was sufficient to preserve focal complexes and support productive lamellipodial protrusion.

### Redistribution of NMII after Blebbistatin Treatment

**Figure 2:**
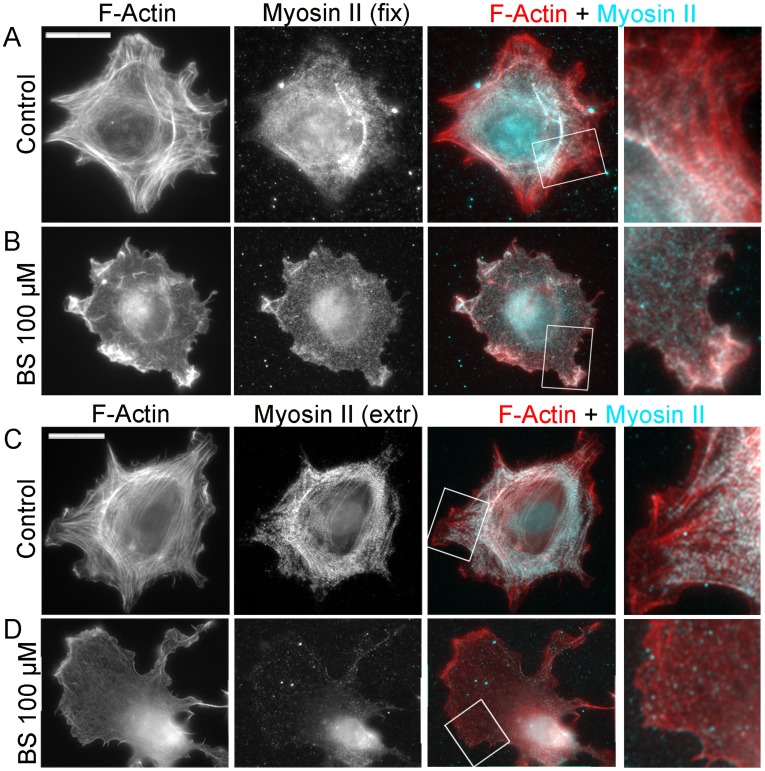
Blebbistatin causes redistribution of NMII to the cell periphery and its dissociation from the cytoskeleton. Fluorescence microscopy of phalloidin-stained F-actin and immunostained NMII in untreated (A,C) and 100 µM blebbistatin (BS)-treated (B,D) cells either directly fixed (A,B) or pre-extracted with detergent before fixation (C,D). Boxed regions are zoomed in right panels. Scale bars, 20 µm.

Immunofluorescence staining of NMII in control cells, either directly fixed (Figure 2A) or pre-extracted with a detergent (Figure 2C), showed a striated pattern in stress fibers and scattered puncta in the lamella, as reported previously [18]. After treatment with 100 µM blebbistatin (Figure 2B), the NMII distribution in directly fixed cells became more diffuse with distinct enrichment in ruffles in 76% of cells (N = 34) versus 2% in untreated cells (N = 48). Similar, but less prominent redistribution of NMII toward the cell periphery was observed in cells treated with 75 µM blebbistatin (Figure S1E). However, NMII staining in blebbistatin-treated cells was largely lost after pre-extraction with detergent (Figure 2D and S1D). Thus, cells treated with 100 µM or 75 µM blebbistatin retained, respectively, only 12.3±4.2% (N = 21 cells) and 16.8±4.5% (N = 18) of the NMII immunoreactivity, as compared to untreated cells (100±2.0%, N = 31).

Dissociation of NMII from the cytoskeleton may result from the detachment of intact bipolar filaments, because blebbistatin locks NMII in an ATP-bound state with weak actin-binding affinity, or from filament depolymerization, or both. To distinguish these possibilities, we performed gradient centrifugation of cytosolic fraction of cell lysates obtained from untreated or blebbistatin-treated cells (Figure S2B). Probing the gradient fractions with NMII antibody showed that NMII species reproducibly separate into two peaks, one in the range of ∼6S–10S, thus corresponding to NMII monomers [42], and the other >16S, likely corresponding to NMII filaments [43]. The normalized amounts of NMII in both peaks were similar in untreated and blebbistatin-treated cells (Figure S2B) suggesting that blebbistatin causes both detachment of intact NMII filaments from the cytoskeleton and their depolymerization, so that the ratio between these two species remains roughly the same.

**Figure 3:**
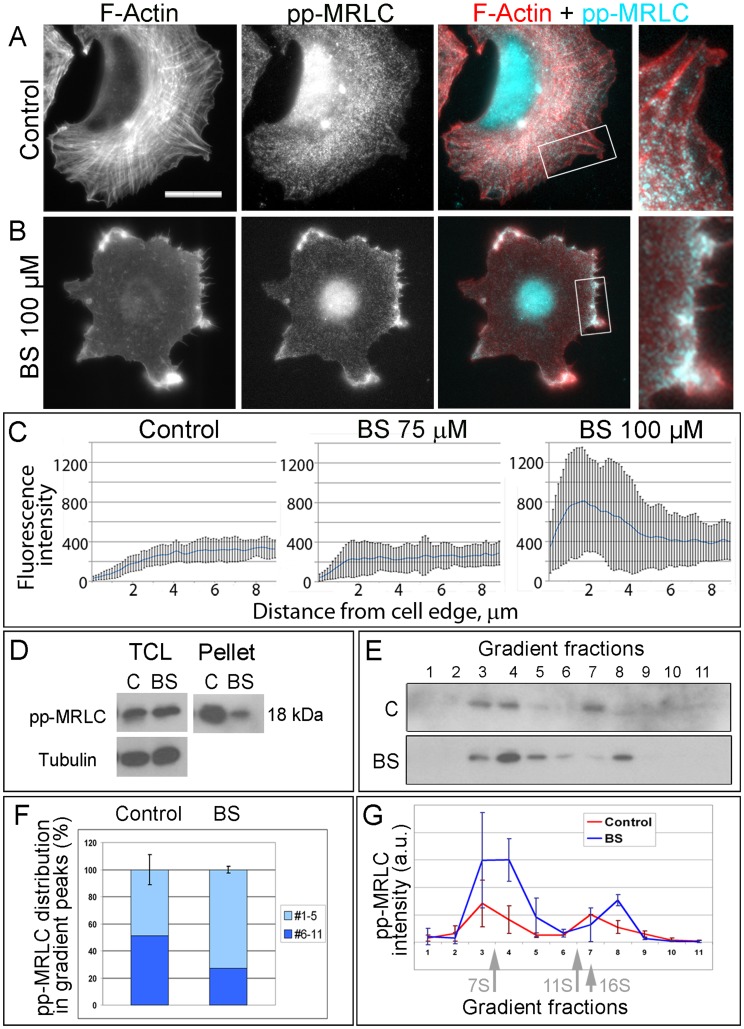
Blebbistatin causes enrichment of pp-MRLC in protrusions. (A,B) Fluorescence microscopy of phalloidin-stained F-actin and immunostained pp-MRLC in untreated (A) and 100 µM blebbistatin (BS)-treated (B) cells. Boxed regions are zoomed in right panels. Scale bar, 20 µm. (C) Fluorescence intensity profiles of pp-MRLC immunostaining in peripheral regions of untreated or blebbistatin-treated cells. Error bars, SD. (N = 10 cells, 30 linescans). (D) Western blotting with pp-MRLC antibody of total cell lysates (TCL) and pellets of untreated (C) and blebbistatin-treated (BS) cells. Tubulin staining is used as loading control. (E−G) Separation of NMII pools by gradient centrifugation of low speed supernatants from untreated (C, Control) or blebbistatin-treated (BS) cells followed by SDS-PAGE and Western blotting with pp-MRLC antibody. (E) Representative Western blot shows two populations of pp-MRLC with peaks in fractions 3–4 and 7–8 with sedimentation coefficients corresponding to NMII monomers and NMII filaments, respectively. (F) Relative amounts of pp-MRLC in two gradient peaks in untreated (Control) and blebbistatin-treated cells (BS). Cumulative pp-MRLC band intensities in top (#1–5) and bottom (#6–11) gradient fractions are presented as percentage of the total amount of pp-MRLC in respective samples. Error bars, SD (N = 2 experiments). Blebbistatin-treated cell have greater relative amount of pp-MRLC-positive monomers. (G) Average intensities in arbitrary units (a.u.) of pp-MRLC bands in individual fractions plotted against the fraction number. Arrows indicate position of marker proteins: aldolase (7S); catalase (11 S) and ferritin (16S). Error bars, SD (N = 2 experiments).

To determine the activation state of NMII in cells, we stained cells with antibodies recognizing MRLC mono-phosphorylated on Ser19 (p-MRLC) or MRLC double-phosphorylated on Thr18/Ser19 (pp-MRLC). Neither antibody revealed obvious changes in the levels of MRLC phosphorylation after blebbistatin treatment (Figure 3A and B), similar to previous reports [44], [45]. Western blot analysis also showed roughly the same levels of pp-MRLC in total cell lysates of untreated and blebbistatin-treated cells (Figure 3D), whereas a significant loss of pp-MRLC was observed in the pellet fraction of blebbistatin-treated cells, as compared to untreated cells. Similar to total NMII, treatment with 100 µM blebbistatin caused dramatic redistribution of pp-MRLC, but not p-MRLC (not shown), from stress fibers to ruffles (Figure 3A−C) that was seen in 85% of cells (N = 26), as compared to 4% in untreated cells (N = 26). Such redistribution was not as prominent in cells treated with 75 µM blebbistatin (Figure S1E). When we probed the gradient centrifugation fractions with pp-MRLC antibody, we found that the lower peak of NMII had a greater level of immunoreactivity in blebbistatin-treated cells, than in untreated cells (Figure 3E−G). These results suggest that activated, but unpolymerized NMII molecules are present in the cytosol of untreated cells and that this active monomeric NMII pool is significantly increased is blebbistatin-treated cells.

### Structural Organization of the Contractile System in Control Cells

**Figure 4:**
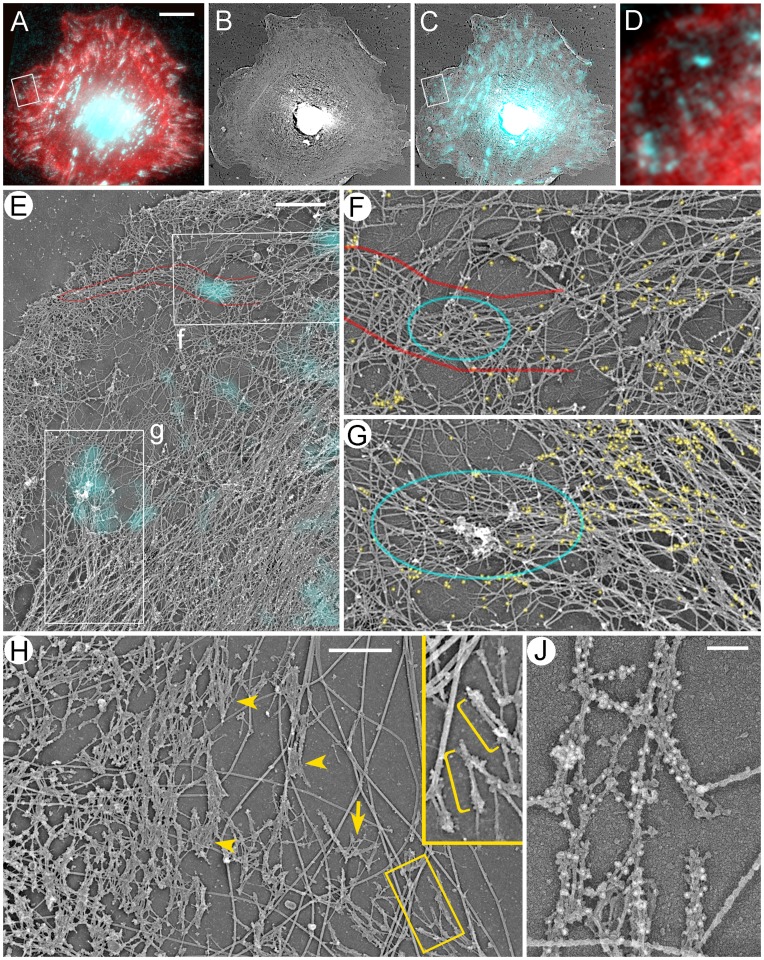
Structural organization of the contractile system in REF52 cells. (A–G) Correlative fluorescence (A, D) and platinum replica EM (B, C,E–G) of a spreading cell fluorescently labeled with phalloidin (red) and vinculin antibody (cyan) and additionally stained with NMII immunogold (F,G, yellow). (A–C) The same cell shown by fluorescence microscopy (A), EM (B), and EM overlaid with vinculin immunofluorescence (C). (D) Enlarged boxed region from A. (E) Enlarged boxed region from C. Upper box (f) marks a focal complex (cyan spot) colocalizing with a filopodial bundle (red outline). Lower box (g) marks a mature focal adhesion at the tip of a stress fiber, which merges with the large transverse bundle of actin filaments (lower right). (F, G) Enlarged upper (F) and lower (G) boxes from E. Red outline in F marks the root of the filopodial bundle. Cyan outlines indicate positions of vinculin fluorescence. Yellow dots mark NMII immunogold particles. (H, J) EM of gelsolin-treated cytoskeleton without (H) or with (J) NMII immunogold labeling. NMII filaments form loose network at the lamellar periphery (arrows) or tight stacks closer to the cell center (arrowheads). Thick fibers are microtubules. Boxed region in H is enlarged in the inset. Individual NMII filaments are marked by yellow brackets. Scale bars, 10 µm (A–C); 1 µm (E, H) and 200 nm (J).

To determine the mutual arrangement of NMII, focal adhesions, and actin filaments at high resolution, we stained detergent-extracted cells with vinculin antibody, imaged them by fluorescence microscopy, and then performed correlative EM of the same cells after immunogold labeling of NMII (Figure 4A–G). In control cells, flat lamellipodia and occasional ruffles were filled with dense dendritic network of actin filaments (Figure 4E), as in other cell types [46]. In the lamella, the cytoskeleton consisting of actin filaments, microtubules, and intermediate filaments was sparse immediately behind the lamellipodium, but became denser in more proximal regions (Figure 4B and E), as in mouse embryo fibroblasts [47]. Correlative EM showed that mature focal adhesions, as expected, corresponded to the tips of straight stress fibers, or were located underneath thick transverse actin arcs (Figure 4C, E, and G), which are commonly found in spreading cells and represent an intermediate stage during stress fiber assembly [21]. Focal complexes in the lamella colocalized with tips of small actin bundles that were not clearly recognizable by light microscopy, or with slightly denser actin networks, or with proximal regions of filopodial bundles (Figure 4F). Some focal complexes superimposed with the rear of the lamellipodial actin network.

Immunogold-labeled NMII was enriched in stress fibers and transverse arcs and also formed patches in the lamella (Figure 4F and G), likely corresponding to clusters of NMII filaments [18]. Gold particles were usually absent in regions corresponding to focal adhesions or focal complexes, but could be seen at a distance from them (Figure 4F and G). To see NMII filaments directly, we removed actin filaments by treating detergent-extracted cells with an actin-severing protein gelsolin [48], [49]. Bipolar NMII filaments in such preparations were recognizable by their dumbbell shape and the characteristic length of ∼300 nm (Figure 4H). Immunogold staining confirmed their identity as NMII filaments (Figure 4J). In the proximal lamella, NMII filaments frequently formed large stacks of parallel NMII filaments that might be further connected into complex networks (Figure 4H). These NMII filament sets likely corresponded to stress fibers or transverse convex arcs. The peripheral lamella usually contained smaller NMII filament assemblies, such as clusters, stacks, chains and some individual NMII filaments.

### Inhibition of NMII Disrupts Actin Cytoskeleton Organization

The organization of the contractile system described in the previous section allowed us to investigate structural aberrations caused by blebbistatin treatment. EM of cells treated with 75 µM blebbistatin revealed that the sparse zone at the lamellipodium-lamella boundary diminished or disappeared (Figure S3A and B). Instead, the relatively normal lamellipodial actin network seamlessly transformed into a remarkably uniform distal lamellar network lacking actin bundles. Interestingly, disappearance of the lamellipodia-lamella boundary in conditions of decreased cell adhesion has been predicted by mathematical modeling [50]. NMII immunogold labeling was greatly diminished in blebbistatin-treated cells, consistent with immunofluorescence data. Remaining gold particles in lamellae were typically uniformly scattered suggesting a loss of NMII filaments. The EM analysis of gelsolin-treated cytoskeletons (Figure S3C and D) confirmed that very few NMII filaments remained after blebbistatin treatment. Notably, these filaments were isolated and did not form stacks or clusters.

**Figure 5:**
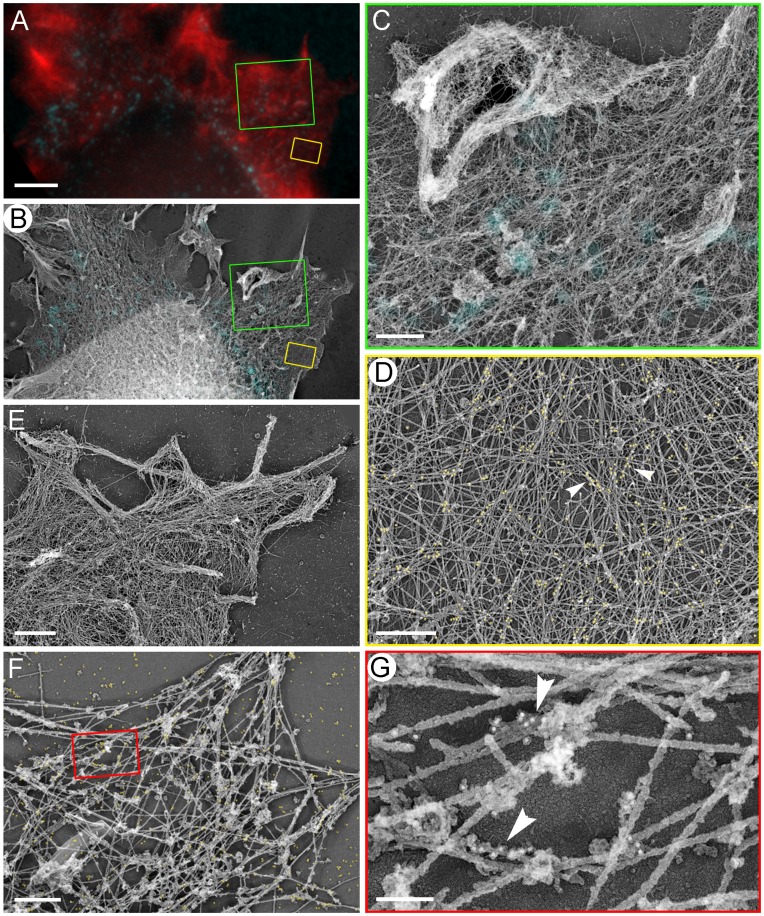
Structural organization of the contractile system in cells treated with 100 µM blebbistatin. (A–D) Correlative fluorescence (A) and EM (B–D) of REF52 cell stained with phalloidin (A, red) and vinculin antibody (cyan) and additionally labeled with NMII immunogold (D, yellow). (A,B) Same cell region shown by fluorescence microscopy (A) and EM overlaid with vinculin immunofluorescence (B). Lighter area at the bottom of B is a reference mark on the coverslip for correlative EM. (C) Enlarged green box from A and B shows a peripheral ruffle (top) and a dorsal protrusion (middle right), as well as disorganized actin network in lamella. Cyan shades indicate weak remaining vinculin fluorescence. (D) Enlarged yellow box from A and B shows actin network in lamella. NMII immunogold particles (yellow dots) sometimes form linear clusters (arrowheads) corresponding to NMII filaments. (E) A peripheral region of another cell showing multiple dorsal and peripheral filopodia. (F, G) EM of gelsolin-treated cytoskeleton after NMII immunogold labeling. (G) Enlarged red box from F. Arrowheads point at few remaining NMII filaments labeled with gold particles (white dots). Thick fibers are microtubules. Scale bars, 5 µm (A); 1 µm (C,E,F); 0.5 µm (D), 200 nm (G).

After treatment with 100 µM blebbistatin (Figure 5), the remaining ruffles and lamellipodia still contained dendritic actin network (Figure 5C), whereas numerous dorsal and peripheral filopodia contained an actin filament bundle (Figure 5E). The actin network in lamellae looked less uniform, than after treatment with 75 µM blebbistatin, possibly, because of significant cell collapse. The immunogold NMII staining was sparse and gold particles were either scattered or formed elongated clusters likely representing remaining NMII filaments (Figure 5D). Accordingly, a few single NMII filaments were detected in gelsolin-treated cytoskeletons (Figure 5F and G). Interestingly, they were frequently associated with microtubules, as if being transported along them, as proposed in [51]. By correlative EM, we were not able to detect any specific actin filament arrangements at weak vinculin-positive sites (Figure 5B and C). Thus, the EM analysis of blebbistatin-treated cells revealed disassembly of actin bundles and dissociation of NMII filaments from the lamellar cytoskeleton.

### Dynamics of Cytoskeleton Reformation after Blebbistatin Washout

**Figure 6:**
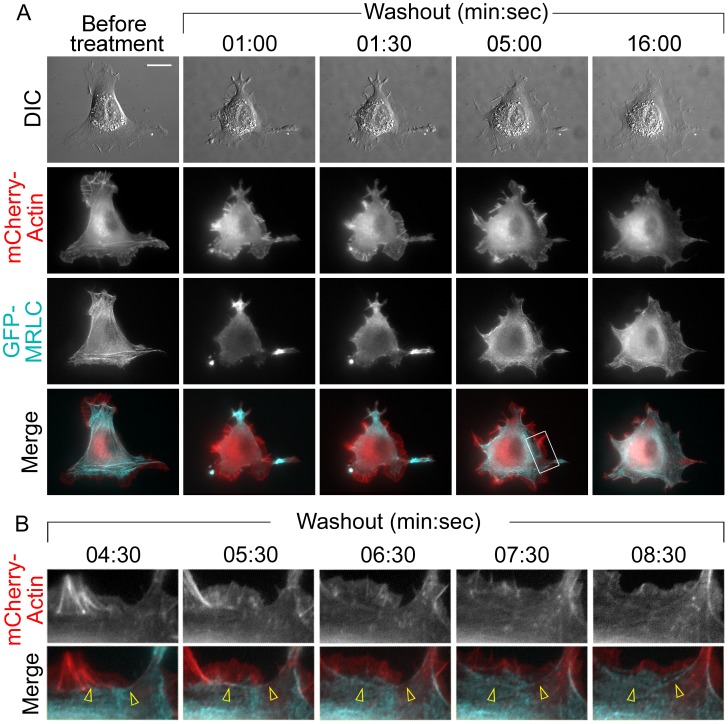
Dynamics of cell recovery after blebbistatin washout. (A) Still DIC and fluorescence frames from the time lapse sequence showing a cell cotransfected with mCherry-actin and GFP-MRLC, treated with 100 µM blebbistatin and washed out of the drug for indicated times. Single images from each channel taken before application of blebbistatin are shown in the first column. Bottom row shows merged images. Scale bar, 10 µm. (B) A part of the time lapse sequence of the boxed region in A shown at greater spatial and temporal resolution to illustrate transformation of filopodial roots (4∶30, arrowheads) to a lateral concave arc at the base of lamellipodium (5∶30) and then to more deeply located stress fibers (6∶30–8∶30).

The acute recovery of the contractile system after removal of blebbistatin was first analyzed by time-lapse fluorescence microscopy of REF52 cells cotransfected with mCherry-actin and GFP-MRLC (Figure 6 and Video S1). Since blebbistatin is photounstable [52], [53], we kept cells in dark during blebbistatin treatments and began time-lapse imaging immediately (∼1 min) after blebbistatin washout. At the onset of imaging, cells displayed vigorous lamellipodial protrusion, which continued for several minutes, then decreased and stabilized at a lower level by ∼15 min of washout (Figure 6A). Actin bundles were first detectable as small arcs along concave cell edges after ∼ 3–5 min of washout. They were frequently formed by merging of filopodial roots (Figure 6B). Straight stress fibers began to form a few minutes later from these concave arcs which moved retrogradely becoming thicker and straighter on the way (Figure 6B). Other bundles formed in the cell interior without obvious relation to lateral arcs.

The GFP-MRLC fluorescence at the beginning of blebbistatin washout was enriched in the peripheral cell processes and ruffles, whereas the cell body was relatively dim (Figure 6A). After washout, GFP-MRLC quickly moved away from the cell periphery and redistributed over the cell body. By 2–3 min of recovery, very little fluorescence remained in pre-existing processes. GFP-MRLC was also undetectable in newly formed lamellipodia. Instead, numerous GFP-MRLC puncta appeared in lamellae, especially in lateral concave actin arcs, and gradually formed more distinct lines corresponding to straight actin stress fibers (Figure 6B). By 15 min of washout, the overall pattern of actin and NMII distribution in most cells was roughly similar to that in untreated cells.

**Figure 7:**
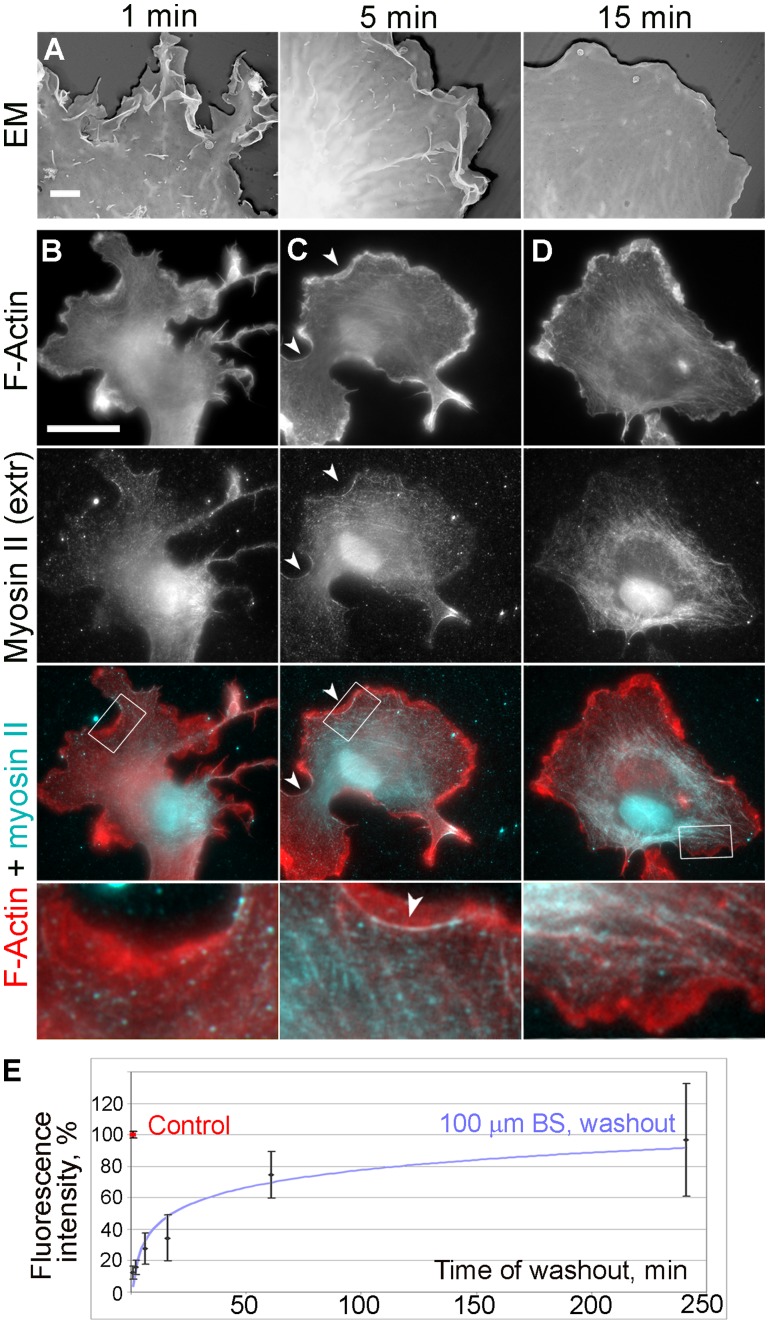
Restoration of lamellipodia and NMII organization after blebbistatin washout. (A) Cell surface topography revealed by platinum replica EM of non-extracted cells washed out of 100 µM blebbistatin for 1, 5, or 15 min. Scale bar, 2 µm. (B–D) Fluorescence microscopy of phalloidin-stained F-actin and immunostained NMII in cells washed out of 100 µM blebbistatin for 1, 5, or 15 min. Scale bar, 20 µm. Boxed regions are zoomed in the bottom row. Arrowheads in C point to concave lateral arcs. (E) Restoration of NMII association with the cytoskeleton in cells recovering from treatment with 100 µM blebbistatin. Immunofluorescence intensity of NMII in detergent-extracted cells is plotted against time after blebbistatin washout. Data are shown as percentage of NMII intensity in untreated cells. Error bars, SD (N = 13–31 cells).

We also analyzed cells fixed at different time points after washout of 100 µM blebbistatin (Figure 7–9). Based on live cell imaging, we selected three time points: 1 min, when active lamellipodial protrusion begins; 5 min, when lamellipodia reach their maximal size and lateral concave actin arcs are formed; and 15 min, when the process of recovery is apparently completed. EM analysis of non-extracted cells demonstrated gradual transition from highly complex cell surface topography after 1 min of washout to smooth flat lamellipodia at the 15 min time point with an intermediate ruffling morphology after 5 min of recovery (Figure 7A). Phallodin staining also revealed quick restoration of lamellipodia already after 1 min washout (Figures 1F and 7B). Conversely, no significant change in phalloidin staining was seen in lamellae after 1 min of recovery, except for rare tiny arcs at some concave cell edges or bases of emerging lamellipodia. After 5 min of recovery, these arcs became more common and additionally, a few thin straight actin bundles appeared in lamellae (Figure 7C). These straight bundles became numerous after 15 min of recovery. Immunostaining with α-actinin (Figure S4) confirmed robust recovery of lamellipodia soon after blebbistatin washout. However, α-actinin was still undetectable along the length of apparently normal stress fibers that were formed in cells undergoing recovery for 15 min. At this time point, α-actinin could be occasionally detected only at the tips of stress fibers, likely corresponding to mature focal adhesions (Figure S4C). Punctate distribution of α-actinin along stress fibers became apparent starting from ∼1 hour after blebbistatin washout (Figure S4D) and acquired a distribution comparable to that in untreated cells by 4 h of washout.

**Figure 8 pone-0040814-g008:**
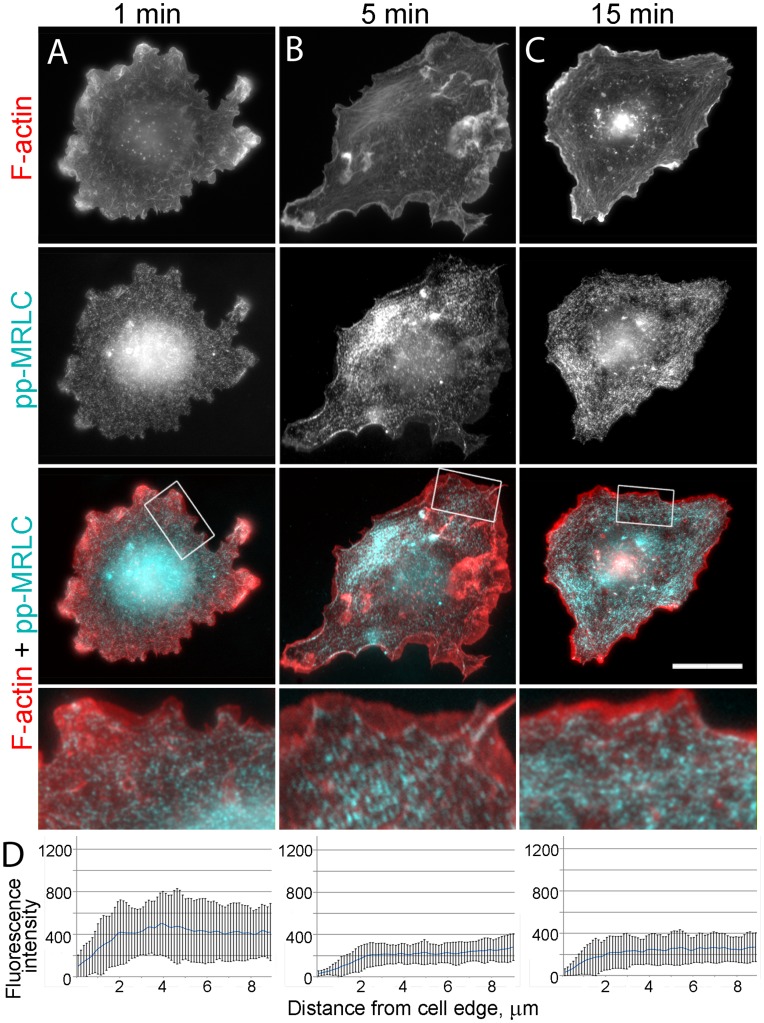
Restoration of pp-MRLC organization after blebbistatin washout. (A–C) Fluorescence microscopy of phalloidin-stained F-actin and immunostained pp-MRLC in cells washed out of 100 µM blebbistatin for 1, 5, or 15 min. Scale bar, 20 µm. Boxed regions are zoomed in the bottom row. (D) Fluorescence intensity profiles of pp-MRLC immunostaining in peripheral regions of cells washed out of 100 µM blebbistatin for 1, 5, or 15 min. Error bars, SD (N = 10 cells, 30 linescans).

**Figure 9:**
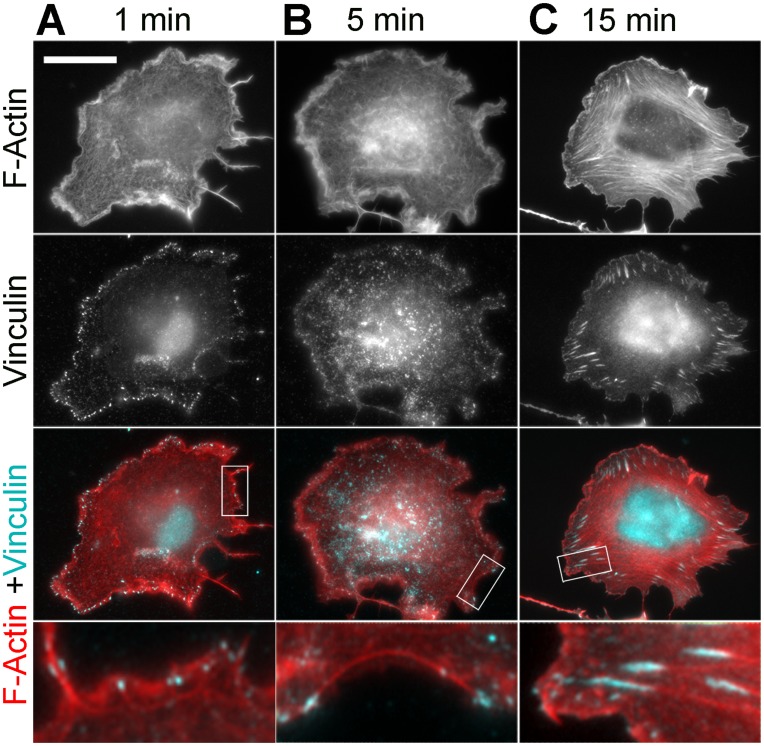
Restoration of focal adhesions after blebbistatin washout. Fluorescence microscopy of phalloidin-stained F-actin and immunostained vinculin in cells washed out of 100 µM blebbistatin for 1, 5, or 15 min. Scale bar, 20 µm. Boxed regions are zoomed in the bottom row.

Immunostaining of directly fixed (not pre-extracted) cells with NMII antibody showed that NMII fluorescence gradually disappeared from the cell periphery after blebbistatin washout and became distributed in the cytoplasm (Figure S5). For better visualization of the cytoskeleton-associated NMII, we immunostained detergent-extracted cells at different time points after blebbistatin washout (Figure 7). The intensity of cytoskeleton-associated NMII immunofluorescence increased slowly after blebbistatin washout. After 1 min, its level barely reached statistical significance (p = 0.047) compared to that in blebbistatin-treated cells. At later time points, the increase in the cytoskeleton-associated NMII fluorescence became statistically significant (p<0.0001), but approached control levels only by ∼ 4 hrs after blebbistatin washout (Figure 7F). By visual inspection, NMII was still largely absent from the cytoskeleton after 1 min washout. Only infrequently, NMII can be seen in lateral concave arcs at 1 min time point (Figure 7B), but such localization became prominent by 5 min washout (Figure 7C). NMII spots also appeared in lamellae at 5 min time point, sometimes colocalizing with thin actin bundles. At 15 min, the pattern of NMII distribution already appeared largely normal despite its lower amount. Specifically, NMII clearly accumulated in straight stress fibers with an occasional striated pattern. Staining with the pp-MRLC antibody (Figure 8) showed a gradual loss of fluorescence from lamellipodia with its concomitant increase in the lamella during recovery from blebbistatin treatment over a span of 15 min.

Staining with vinculin antibody (Figure 9) showed that multiple focal complexes were formed along the cell edge already after 1 min recovery. Many focal complexes were present along filopodia or microspikes or at the base of lamellipodia (Figure 9A). If concave arcs were present, focal complexes were usually located at their tips or along the length. At 5 min time point, peripheral focal complexes were still prominent, but many focal complexes also appeared in lamellae. The larger and brighter focal complexes usually colocalized with the tips of concave actin arcs (Figure 9B). By 15 min of recovery, mature elongated focal adhesions were found at the tips of straight stress fibers in lamellae (Figure 9C). At this point, some cells were indistinguishable from untreated cells, whereas other cells had less organized stress fibers and focal adhesions.

Together, the light microscopic analysis revealed that the early stages of the contractile system restoration involve quick and nearly simultaneous reformation of focal complexes, lamellipodia, and lateral concave arcs. It was followed by appearance of NMII accumulations in the concave arcs. The mature focal adhesions and straight stress fibers emerged from these precursor structures, but semi-sarcomeric patterns of NMII and α-actinin localization became visible only after 1 h of blebbistatin washout and was apparently complete by 4 h of recovery.

### Structural Reformation of the Contractile System

**Figure 10:**
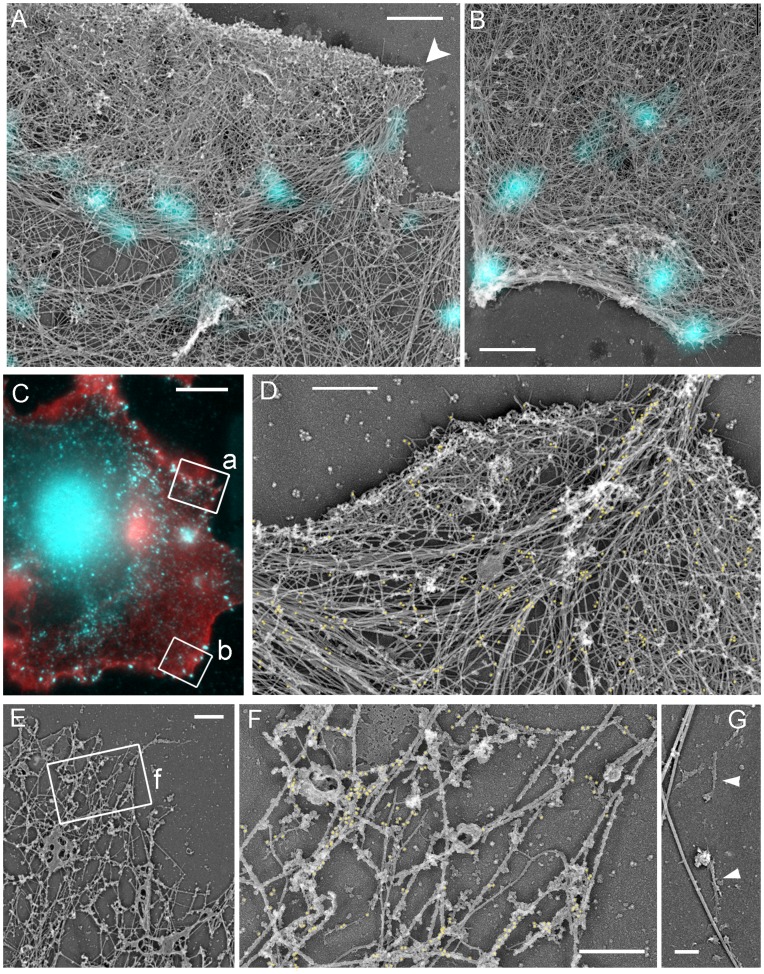
EM of cells recovering for 1 min after washout of 100 µM blebbistatin. (A–C) Correlative fluorescence (C) and EM (A,B) of REF52 cell fluorescently labeled with phalloidin (C, red) and vinculin (cyan) antibody. Focal complexes (cyan spots) colocalize with concave arc-shaped bundles of long actin filaments at the base of lamellipodium (A) or at the cell edge (B). The concave arc in A is continuous with a filopodial bundle terminating at the lamellipodial edge (arrowhead). Panels A and B correspond to boxed regions a and b, respectively, in panel C. (D) Immunogold staining of NMII. Yellow dots mark gold particles that are evenly scattered and not abundant. (E–G) EM of gelsolin-treated cytoskeleton with (E,F) or without (G) NMII immunogold labeling. Boxed region in E is enlarged in F. Yellow dots in E mark NMII immunogold particles. Arrowheads in G point to occasional NMII filaments. Scale bars, 1 µm (A,B),10 µm (C), 0.5 µm (D,F), 1 µm (E) and 200 nm (G).

EM was used to examine cells recovering from blebbistatin treatment at higher resolution. Already after 1 min washout of 100 µM blebbistatin (Figure 10), many lamellipodia were seen in the cells. The lamellipodium-lamella boundary was clearly demarcated by a region of the sparse cytoskeleton (Figure 10A). A small concave arc of long bundled actin filaments was frequently present at the base of lamellipodia, even though such arcs were not clearly visible by light microscopy at this time point (Figure 10A–C). The arcs terminated at the cell edge and were continuous with filopodium-like bundles (Figure 10A). Concave arcs were also found along the cell edges without lamellipodia (Figure 10B). They might represent even earlier stages of recovery. Correlative EM of cells subjected to vinculin immunofluorescence staining showed that focal complexes were frequently associated with filopodia and lateral concave arcs (Figure 10A). Concave arcs without lamellipodia usually associated with focal complexes only at their tips (Figure 10B), whereas arcs at the base of lamellipodia frequently overlapped with multiple focal complexes (Figure 10A). Focal complexes could also be seen at the base of lamellipodia without concave arcs. According to NMII immunoEM, gold particles were still quite rare after 1 min of recovery, even in concave arcs and roots of filopodia associated with focal complexes (Figure 10D). NMII filaments were also rare in gelsolin-treated cytoskeletons, where they localized individually or formed short chains (Figure 10E and F).

**Figure 11:**
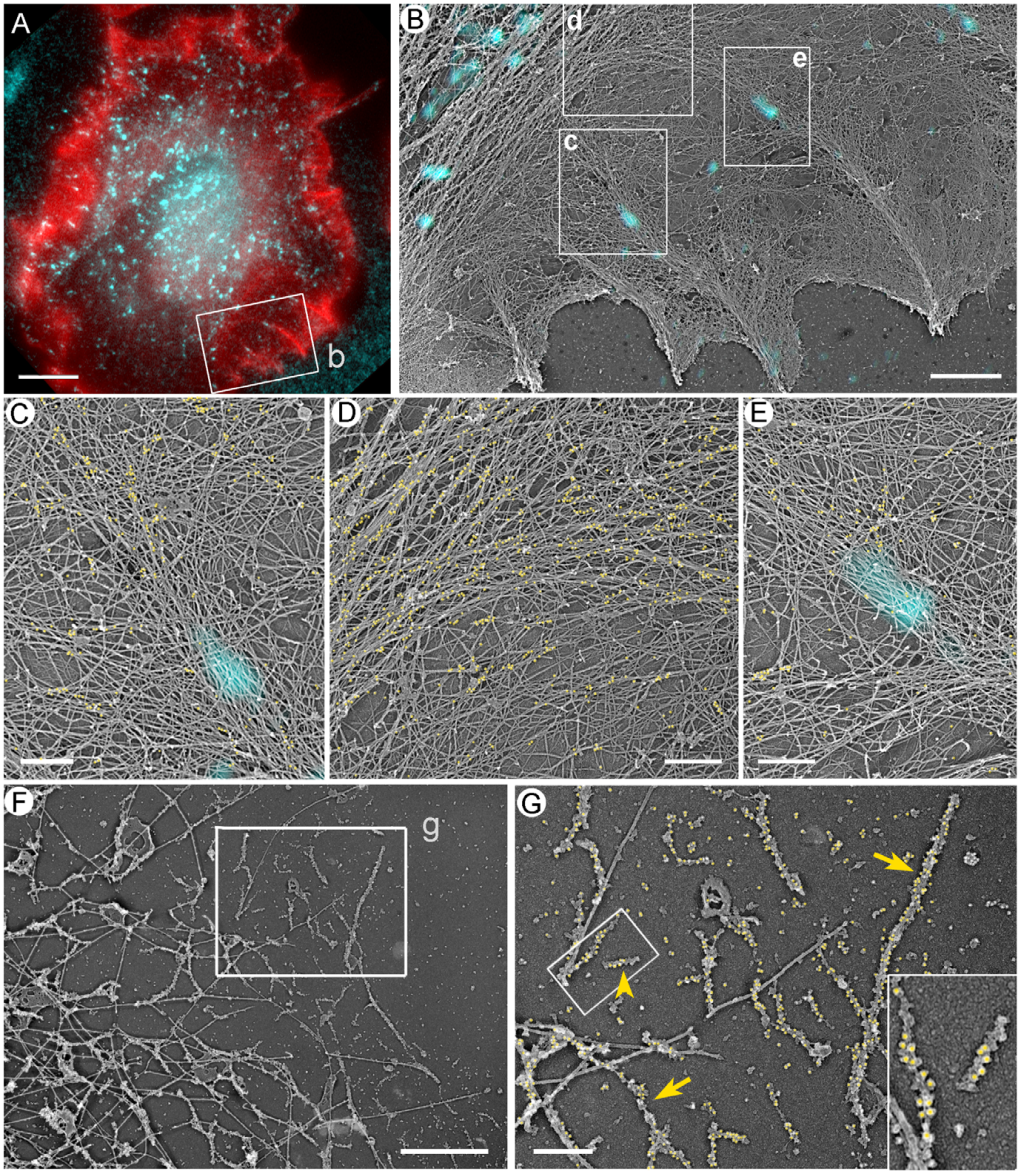
EM of cells recovering for 5 min after washout of 100 µM blebbistatin. (A–E) Correlative fluorescence (A) and EM (B–E) of REF52 cell stained with phalloidin (A, red) and vinculin antibody (cyan) and additionally labeled with NMII immunogold (C–E, yellow). (B) EM of boxed region in A overlaid with vinculin immunofluorescence in cyan. Focal complexes (cyan spots) colocalize with roots of filopodial bundles (boxes c and e) and large transverse bundle of actin filaments (upper left). Roots of filopodial bundles bend and merge with this large transverse bundle (box d). (C–E) Enlarged boxes from B labeled by corresponding letters. Cyan spots mark position of focal complexes. (F,G) EM of gelsolin-treated cytoskeleton after NMII immunogold labeling (G, yellow). Boxed region from F is enlarged in G. Arrows point to longitudinal chains of NMII filaments; arrowhead points to an individual NMII filament. Boxed region in G is enlarged in inset to show a short chain of two gold-labeled NMII filaments and an individual NMII filament (to the right). Scale bars, 10 µm (A); 2 µm (B,F); and 0.5 µm (C–E, G).

By 5 min of recovery (Figure 11), lamellipodia became very broad and frequently contained internal filopodial bundles. Peripheral concave arcs became larger and localized deeper in the lamella (Figure 11B), as compared to 1 min washout, and could be composed of several smaller bundles, some of which originated from filopodial roots. Other parts of lamella contained multiple thin straight bundles with predominant radial orientation (not shown). Individual filaments within all these bundles were long and spanned large distances. By correlative EM, vinculin-positive focal complexes often colocalized with filopodial roots and concave arcs, although they were also present in peripheral lamellae (Figures 11C, E and S6) and under large transverse arcs in deep lamellar regions (Figure 11B).

ImmunoEM showed increased NMII accumulation in concave arcs (Figure 11D), straight bundles (Figure S6), and some proximal filopodial roots (Figure 11C and S6D), whereas distal regions of filopodial bundles remained unlabeled, as typical for filopodia. Small clusters of gold particles were also scattered in lamella (Figure 11C–E and S6C,D). In gelsolin-treated cytoskeletons (Figure 11F and G), chains of longitudinally connected bipolar filaments were a dominant feature of NMII organization. Some chains were relatively thick, suggesting lateral association of NMII filaments in addition to their longitudinal interaction. However, stacks of NMII filaments characteristic for control cells were not detected. As in blebbistatin-treated cells, NMII filaments or their chains were frequently bound to microtubules.

**Figure 12:**
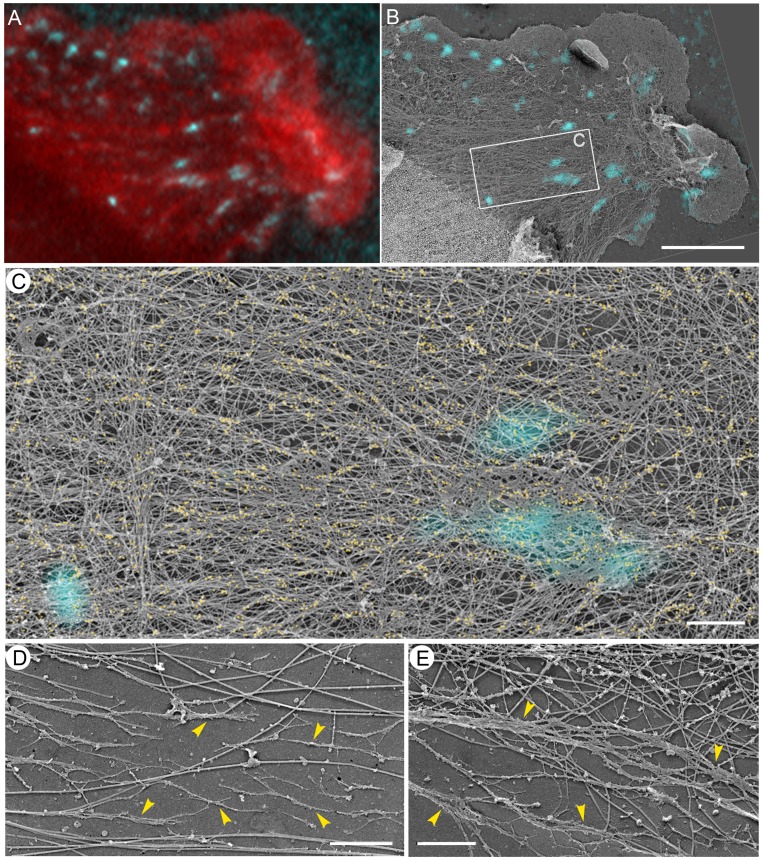
EM of cells recovering for 15 min after washout of 100 µM blebbistatin. (A–C) Correlative fluorescence (A) and EM (B,C) of REF52 cell stained with phalloidin (A, red) and vinculin antibody (cyan) and additionally labeled with NMII immunogold (C, yellow). (A, B) Same cell region shown by fluorescence microscopy (A) and by EM overlaid with vinculin immunofluorescence (B). Focal complexes are located at the bases of lamellipodia (upper left and lower right) and in the body of lamella. Mature focal adhesions (center) colocalize with actin bundles (box). (C) Enlarged box from B. NMII immunogold particles form multiple elongated clusters aligned with actin bundles. (D,E) EM of gelsolin-treated cytoskeleton. Arrowheads point to longitudinal chains of NMII filaments at cell periphery (D) or cell interior (E). Scale bars, 5 µm (A,B) and 1 µm (C–E).

By 15 min of recovery, the cytoskeletal organization of cells was already largely similar to that of control untreated cells, but stress fibers were on average thinner and less abundant and mature focal adhesions were less frequent than in control cells (Figure 12). The main difference, however, was in the organization of NMII filaments in gelsolin-treated samples (Figure 12D and E). NMII filaments still predominantly formed longitudinal chains, although thicker than after 5 min washout, but not stacks, suggesting that the formation of the contractile system was not finished at this time point. Together, these data show that recovery of lamellipodia, focal complexes, and lateral actin arcs precedes reformation of NMII filaments and that filopodial bundles and their derivatives, concave arcs, are the earliest structural precursors of the forming contractile system.

### Actin Polymerization Contributes to Reformation of Lamellipodia and Stress Fibers

We used latrunculin A, an actin monomer-sequestering drug, to test whether actin polymerization is required for restoration of the contractile system after blebbistatin washout. As shown previously, low concentrations of latrunculin A inhibit lamellipodia in REF52 cells, but do not visibly affect stress fibers [35]. Here, treatment of control cells with latrunculin A (0.5 µM for 30 min) caused disappearance of lamellipodia and most focal complexes, whereas mature focal adhesions and large actin bundles were preserved (Figure S7). Simultaneous treatment with 75 µM blebbistatin and 0.5 µM latrunculin A caused loss of both lamellipodia and focal complexes in cells, similar to effects of 100 µM blebbistatin alone (Figure S7A). After washout of blebbistatin in the presence of latrunculin A (Figure S7B), no significant lamellipodium formation was observed up to 15 min of recovery. Importantly, focal complexes appeared all over the cell only 15 min after washout simultaneously with very thin actin bundles, indicating a severe delay in the contractile system recovery in the absence of actin polymerization.

## Discussion

In this study, we used the blebbistatin treatment-and-washout approach to investigate structural mechanisms of the contractile system assembly and the roles of NMII in this process. Although blebbistatin is usually considered as a specific inhibitor of NMII in nonmuscle cells, it should be noted that not all members of the huge myosin superfamily have been tested regarding their sensitivity to blebbistatin, leaving a possibility that other myosins may be also sensitive to blebbistatin. Therefore, although the effects of blebbistatin observed in this study most likely result from inhibition of NMII, we cannot completely exclude a possibility of involvement of other myosins. Although a relatively high blebbistatin concentration of 100 µM may cause a concern regarding specificity of its effects, our results with inactive enantiomer of blebbistatin argue against grossly adverse effects of the drug. Notably, the same 100 µM concentration has been also widely used by many labs [19], [26], [28], [29], [30], [31], [32], [33], [54]. The need for high blebbistatin concentration to achieve complete inhibition of NMII in vivo, as compared to in vitro experiments, may be explained by the fact that blebbistatin has high affinity for ATP-bound NMII and much lower affinity for ADP-NMII [55], whereas NMII has high ADP affinity and slow ADP release, especially under load [56]. Therefore, the slow-cycling NMII species that generate isometric tension in established stress fibers may be less sensitive to blebbistatin. Accordingly, REF52 cells that have a robust stress fiber system are relatively resistant to blebbistatin treatment, especially when it concerns thick lateral or posterior stress fibers. However, many of the dramatic effects of 100 µM blebbistatin begin to develop at a lower (75 µM) concentration, suggesting quantitative, but not qualitative differences between these conditions.

Our results showed that (1) a blebbistatin-sensitive myosin, most likely NMII, functions in the formation of lamellipodia and focal complexes; (2) activated, but unpolymerized NMII accumulates at the cell periphery in the absence of NMII motor activity; (3) the soluble pool of activated NMII stimulates formation of focal complexes before visibly assembling into bipolar filaments; (4) filopodial bundles and concave arcs are preferential sites for the initial formation of focal complexes and assembly of NMII bipolar filaments; (5) stress fiber formation proceeds in parallel with the assembly of NMII bipolar filaments that occurs in association with anchored actin bundles; and (6) recruitment of α-actinin and development of the semi-sarcomeric pattern in stress fibers is the last and slow step of the contractile system assembly. We discuss these points below.

### Focal Complexes and Lamellipodia Depend on each Other and NMII

NMII is a well-recognized key player during formation of stress fibers and maturation of focal adhesions. However, we observed here that focal complexes and lamellipodia are also inhibited by blebbistatin and recover in a coordinate manner after drug washout, suggesting that these structures also depend on NMII activity. This idea is supported by other studies that have shown dependence of focal complexes on the cross-linking activity of NMII [10], and inhibition of protrusions [26], [57], [58], [59] and increased ruffling [29] after blebbistatin treatment [29], [58], [59], or RNAi-mediated knockdown of NMIIB [26] or NMIIA [59], or in NMIIB knockout neurons [57]. Since lamellipodia and focal complexes sustain in the presence of a lower blebbistatin concentration, albeit at a lower level, a small amount of NMII activity may be sufficient for their maintenance. This point, as well as activation of Rac upon NMII inhibition [60], [61], may explain preservation of lamellipodia after NMII inhibition observed in some studies [28], [40], [41], [60], [62], [63], [64]. Indeed, knockdown techniques do not achieve complete elimination of NMII and the knockout approach is complicated by the presence of three isoforms of the NMII heavy chain, two of which, NMIIA or NMIIB, are broadly expressed [3], while double knockouts of NMIIA and NMIIB have not been reported to our knowledge. The early embryonic lethality of mice with the individual knockout of NMIIA or NMIIB [65], [66], probably, precludes generation of double knockouts. Although we cannot completely exclude a possibility that another blebbistatin-sensitive myosin is responsible for inhibition of lamellipodia and focal complexes, somewhat similar observations of inhibited cell spreading and protrusive activity after targeted depletion of individual NMII isoforms [26], [57], [59], [60] argues for at least partial role of NMII in these functions.

The presence of the dendritic actin network in ruffles of blebbistatin-treated cells indicates that actin polymerization still occurs, albeit at a lower level, but protrusions cannot attach, suggesting that NMII may support lamellipodia by stimulating the formation of focal complexes (or even nascent adhesions), which then provide traction for lamellipodia [16], [67]. NMII may stimulate focal complexes by activating mechanosensitive molecules in adhesions, as during focal adhesion maturation [4], but at a much lower, possibly, even single-molecule scale. Since actin polymerization also contributes to adhesion initiation ( [10], [15], [16] and this study), a polymerization-driven component of the retrograde flow [68], [69] may also stimulate focal complexes, supposedly, by applying a drag on adhesion receptors. Relative contribution of actin polymerization and NMII activity to retrograde flow varies among cell types, providing another potential explanation of variable sensitivity of their focal complexes and lamellipodia to NMII inhibition in different cells. Thus, lamellipodia of fish keratocytes may sustain treatment with 100 µM blebbistatin [70], [71], because their powerful actin polymerization machinery exerts sufficient drag on mechanosensors to induce adhesions. In contrast, REF52 cells have relatively weak lamellipodial activity, which likely makes their focal complexes highly dependent on NMII.

### Accumulation of Activated Unpolymerized NMII at the Cell Periphery

NMII is typically not detected in lamellipodia. NMII filaments normally begin to form in lamellae, well behind the lamellipodia, and subsequently undergo retrograde flow gradually coalescing into large assemblies [17], [18]. This fact inspired an idea that NMII molecules should be activated in the lamella, where they would immediately polymerize into filaments and start functioning as contractile devices. However, our current data suggest that this model should be revised. Indeed, when the actin-binding and motor activities of NMII are blocked by blebbistatin, the pp-MRLC-bound form of NMII accumulates at the cell periphery suggesting that activation by MRLC phosphorylation occurs close to the leading edge, rather than in the lamella. This idea is attractive because the plasma membrane is a common place to recruit various regulators, whereas it is not so easy to imagine which structure(s) might recruit NMII activators in the lamella. The lack of NMII enrichment in lamellipodia of untreated cells suggests that NMII leaves protrusions shortly after MRLC phosphorylation in a motor- or actin-binding dependent manner. Consistent with this idea, we observed that GFP-MRLC quickly moves out of protrusions after blebbistatin washout. Although restored motor activity is expected to drive NMII toward the membrane, a faster actin retrograde flow may produce its net rearward drift. Notably, enrichment of NMII [72] and pMRLC [26], [73] has been also detected in ruffles of actively protruding cells, supporting our hypothesis of the transient presence of NMII in protrusions.

### Soluble Pools of Activated NMII Promote Focal Complex Formation and Lamellipodial Protrusion

We found that a large fraction of NMII in blebbistatin-treated cells exists in a monomeric, yet MRLC-phosphorylated form, suggesting that NMII filament polymerization is inhibited in these conditions despite ongoing MRLC phosphorylation. Furthermore, our data show that focal complexes and lamellipodia begin to recover after blebbistatin washout before a significant increase in the cytoskeleton-associated pool of NMII and detectable formation of NMII filaments. These findings suggest that a soluble pool of active NMII is sufficient for the initial recovery. At present, we cannot distinguish whether this activity belongs to activated NMII monomers that become enriched in blebbistatin-treated cells or to small oligomers that are quickly formed after blebbistatin washout, while remaining soluble. However, the enrichment in lamellipodia of molecules that negatively regulate NMII polymerization at the heavy chain level [58], [72], [74], [75] favors the former possibility. Indeed, in combination with our hypothesis that NMII may be activated by MRLC phosphorylation in protrusions, these data suggest that active NMII monomers should be a dominant, but transient, population of NMII in lamellipodia. Although activated NMII monomers are enriched in blebbistatin-treated cells, they are also present in untreated cells, suggesting that they may have specific functions in normal conditions. Accordingly, monomeric NMIIA has been shown to stimulate secretion of lytic granules in natural killer cells [76]. Together, these data suggest that monomeric motor-active NMII may be a genuine functional species in cells, thus contrasting the general assertion that NMII functions exclusively in the form of bipolar filaments.

The exact mechanism of how unpolymerized NMII can stimulate focal complex formation remains to be fully understood. Most likely, this mechanism involves generation of small traction forces [16] that would stabilize very dynamic nascent adhesions and transform them into focal complexes [10]. For example, by physically interacting with phospholipids [77] or integrins in an actin-independent manner [78], NMII can generate small traction forces during leading edge protrusion. Alternatively, monomeric NMII may use its two heads to exert stress on adhesion receptors by cross-linking or pulling on attached adjacent actin filaments [10], [79].

### Tension-dependent Polymerization of NMII Filaments

After blebbistatin removal, NMII quickly leaves protrusions with the retrograde flow, and slowly, but steadily polymerizes into bipolar filaments in the lamella. Initiation of NMII polymerization in the lamella may be explained by release from the inhibitory heavy chain regulation existing in lamellipodia. However, the NMII filament assembly in lamella is not uniform, but shows a preference for filopodial bundles and lateral concave arcs, suggesting a positive regulation at these locations. Since filopodial bundles and concave arcs showed preferential association with focal complexes at the earlier stage of the recovery, they should provide greater resistance to NMII-mediated pulling force and thus be under greater tension. Therefore, we interpret preferential assembly of NMII filaments in association with these actin bundles as a tension-dependent process. It is analogous to the previously reported tension-dependent accumulation of NMII at the strained sites on the Dictyostelium plasma membrane [80], [81] or in the epithelial layer of Drosophila embryos [82]. In these studies, it was hypothesized that NMII assembly is regulated by tension-dependent MRLC phosphorylation. However, this hypothesis is not supported by the data that the level of MRLC phosphorylation remains the same ( [44], [45] and our study), or even increases [83], [84], in the presence of blebbistatin. A more likely explanation is based on the finding that myosin II has a preference for binding stretched conformation of actin filaments relative to relaxed filaments [85]. NMII binding to particular subsets of actin filaments may also be enhanced by specific tropomyosin isoforms [22].

We speculate that long actin filaments in filopodial bundles have more chances to capture several NMII molecules, which would collectively exert enough force to induce focal complexes in association with these bundles. The resistance of focal complexes, in turn, generates stretched filaments, which would capture even more NMII molecules due to increased affinity. A high local concentration of NMII molecules on these bundles can then promote bipolar filament assembly at the sites of increased tension. Lateral concave arcs located at the base of a lamellipodium may additionally experience dragging forces from the retrogradely flowing actin network, which would contribute to generation of focal complexes, tense filaments, and NMII polymerization. At the later stages of recovery from blebbistatin, focal complexes and thin nascent stress fibers also appear in the lamellar interior, possibly, following a similar tension-dependent mechanism when activated NMII molecules arrive to these locations. Although a significant fraction of soluble NMII in blebbistatin-treated cells is present in the filamentous form, our data are not consistent with an idea that detached bipolar filaments simply rebind the actin cytoskeleton after washout of the drug, because in such case, we would observe very fast recovery of NMII association with the cytoskeleton and appearance of numerous NMII filaments in the EM samples. Instead, our results indicate that NMII filaments are likely assembled from available activated monomers, as also shown for tension-dependent myosin accumulation in Dictyostelium [80].

Tension-dependent assembly of NMII filaments suggests that upon relaxation, such as in the presence of blebbistatin, this load-dependent regulation might conversely cause disassembly of unloaded filaments. Consistent with this idea, we found that partial depolymerization accompanies detachment of NMII filaments after blebbistatin treatment, so that the ratio between monomeric and polymeric NMII pools remains unchanged, as compared to untreated cells. If NMII solubilization occurred only through detachment of intact NMII filaments, we would have observed an increased filament-to-monomer ratio in the cytosols of blebbistatin-treated cells.

Thus, we propose a two-step mechanism of NMII activation. In the first step, NMII is activated in protrusions by MRLC phosphorylation, but its polymerization is locally inhibited by heavy chain regulators. In the second step, polymerization of activated NMII is allowed in the lamella and promoted by a tension-dependent mechanism. This idea significantly revises the current belief that MRLC phosphorylation and NMII filament assembly occur simultaneously.

### Assembly of the Mature Contractile System

By investigating the assembly of the contractile system virtually from scratch, we have determined that filopodial bundles and concave arcs made of filopodial roots are the earliest stress fiber precursors. Previous studies on the stress fiber assembly under normal culture conditions also showed that stress fibers originate from leading edge protrusions, filopodia [19], [20] or lamellipodia [21], [22], and this process frequently involves intermediate formation of large transverse arcs [21], [86]. Here, we additionally provide high resolution structural information about the contractile system assembly at the level of individual filaments. Thus, our EM data show that even the youngest stress fibers are composed of long actin filaments that span large distances along the length of the stress fiber equivalent to several semi-sarcomeric units. Considering the origin of stress fibers from filopodial bundles, the presence of long filaments is not particularly surprising. However, this finding is not consistent with a model suggesting that stress fiber formation occurs by endwise association of short actin bundles [21], [22]. Possibly, light microscopic images lacking sufficient resolution were mis-interpreted in these studies, or cells can use different mechanisms depending on physiological conditions.

Our data also reveal that the semi-sarcomeric pattern of alternating NMII and α-actinin stripes [18] forms very late in the course of the stress fiber reformation after blebbistatin washout. Specifically, normally sized stress fibers associated with apparently mature focal adhesions are formed within minutes after blebbistatin washout. However, these fibers lack α-actinin and contain NMII filaments as continuous chains, instead of discontinuous stacks, whereas the formation of a semi-periodic arrangement of NMII and incorporation of α-actinin into stress fibers takes hours after blebbistatin washout. A delayed recruitment of α-actinin, as compared to NMII, after blebbistatin washout was also observed in U2OS cells [87]. In contrast, constitutive formation of stress fibers in the same cell type involves immediate development of the semi-periodic NMII pattern and incorporation of α-actinin [21], [22]. This difference may be explained by the presence of pre-existing structures in untreated cells that could promote early maturation of stress fibers. However, disassembly of the pre-existing contractile system by blebbistatin helped us to reveal that the process of stress fiber formation includes a maturation step that is separate from and subsequent to the initial assembly of stress fibers.

### Conclusion

**Figure 13:**
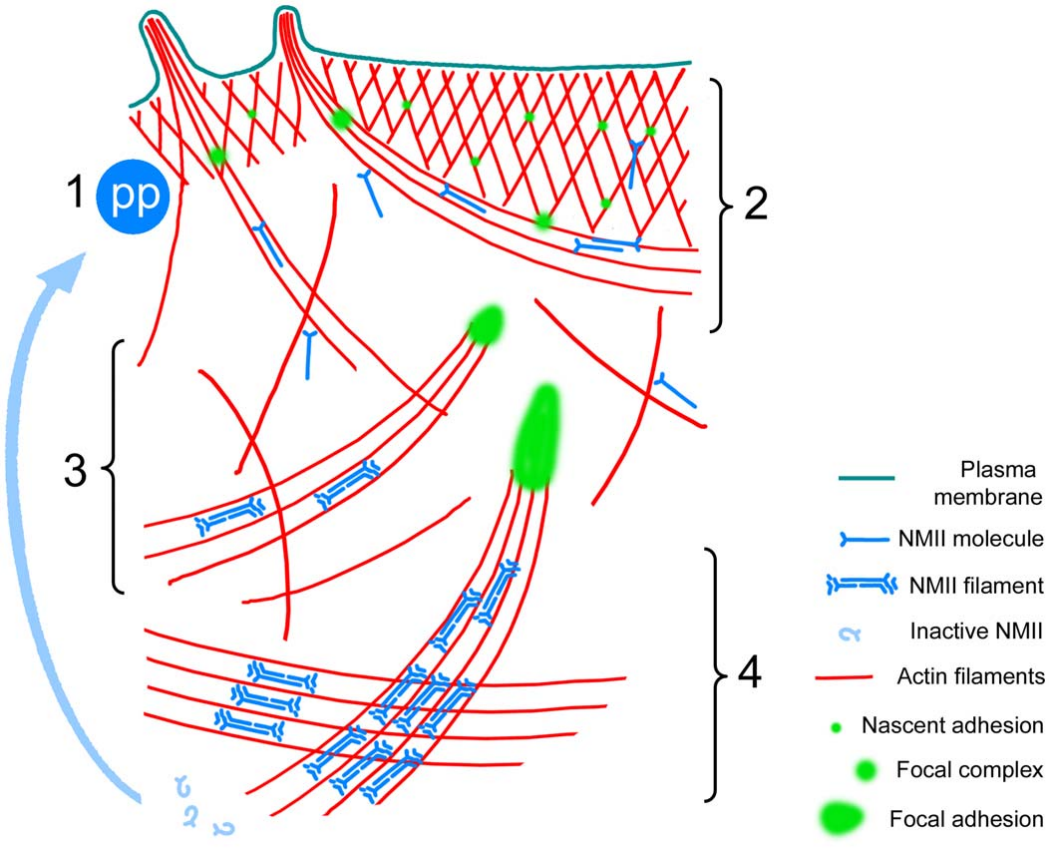
Model for NMII functions during contractile system assembly. (1) *NMII activation*: Inactive NMII molecules diffuse to lamellipodia, where they are activated by double phosphorylation of MRLC. (2) *Focal complex formation:* Active unpolymerized NMII molecules bind actin filaments in lamellipodia and undergo the retrograde flow with them. If two NMII heads bind different actin filaments, one of which is anchored to a nascent adhesion, the resulting strain stabilizes the nascent adhesion and promotes its transformation to a focal complex. Long filaments in filopodia can encounter more NMII molecules increasing a probability of focal complex formation under filopodial bundles. (3) *Assembly of NMII filaments:* NMII molecules pulling on actin filaments attached to focal complexes experience a greater load, which triggers tension-dependent NMII polymerization at these sites. (4) *Stress fiber formation:* Multivalent NMII filaments exert large forces sufficient to promote maturation of focal complexes to focal adhesions. They also cross-link and align disordered actin filaments into bundles producing stress fibers. However, additional events, such as recruitment of α-actinin, are needed for the formation of semi-sarcomeric pattern in stress fibers.

Based on our results, we propose a model for the mechanism of contractile system assembly and the role of NMII in this process (Figure 13): (1) NMII molecules become activated by double phosphorylation at the leading edge. (2) While still in the monomeric form, they generate small traction forces in lamellipodia that stabilize nascent adhesions and promote their transition to focal complexes. This transition is favored at sites of greater mechanostimulation, such as filopodial bundles or lamellipodial bases. Stabilized nascent adhesions and focal complexes allow for sustained lamellipodia protrusion, which in turn supports constant production of new nascent adhesions. (3) Greater resistance of focal complexes to the NMII-generated pulling force activates load-dependent mechanism of NMII polymerization leading to assembly of NMII bipolar filaments at the sites of high tension, namely, along actin filaments attached to focal complexes. (4) NMII filaments exert greater forces than individual NMII molecules and promote maturation of focal complexes to focal adhesions. The increasing number of NMII filaments cross-links and aligns disordered actin filaments into bundles producing nascent stress fibers. However, additional events are needed for the stress fiber maturation that leads to the formation of semi-sarcomeric pattern and incorporation of α-actinin.

## Materials and Methods

### Reagents and Constructs

Working concentrations of (−)- and (+)-Blebbistatin (Toronto Research Chemical Inc.) were prepared from 10 mM stock in DMSO. Latrunculin A (Calbiochem) was prepared from 2 mM stock solution in DMSO. Fluorescently labeled phalloidin was from Molecular Probes. The following primary antibodies were used: mouse monoclonal α-actinin (Cytoskeleton), mouse monoclonal vinculin (Sigma), rabbit polyclonal NMII from bovine spleen [88], rabbit polyclonal mono- (Ser19) or double- (Thr18/Ser19) phosphorylated MRLC (Cell Signaling Technology, a gift from Dr. C. Chen, University of Pennsylvania). Secondary fluorescently labeled antibodies were from Molecular Probes or Jackson Laboratories. GFP-MRLC [89] was a gift of Drs. T.L. Chew and R. Chisholm (Northwestern University) and mCherry-actin was described previously [90]. All other reagents were from Sigma unless indicated otherwise.

### Cell Culture and Light Microscopy

REF52 rat embryo fibroblasts [18] were cultured in DMEM supplemented with 10% FBS and antibiotics at 37°C and 5% CO_2_. For blebbistatin treatment, cells were plated on glass coverslips and allowed to spread for 30 min; then blebbistatin was added and cells were cultured for additional 2 hours. For washout experiments, after 2 h incubation with blebbistatin, cells were transferred to fresh blebbistatin-free medium, incubated for various periods of time, and fixed.

For fluorescence staining, cells were quickly rinsed with PBS and fixed with 4% paraformaldehyde in PBS either directly or after pre-extraction with 1% Triton X-100 in PEM buffer (100 mM PIPES-KOH, pH 6.9, 1 mM MgCl_2_, and 1 mM EGTA) optionally containing 2% polyethelene glycol (PEG) (MW 35,000) and 2 µM phalloidin. Directly fixed samples were permeabilized with 0.1% Triton X-100 in PBS before staining. For costaining of F-actin, labeled phalloidin was added to the secondary antibody solution. Light microscopy was performed using Eclipse TE2000-U inverted microscope (Nikon) equipped with Plan Apo 100×1.3 NA objective and Cascade 512B CCD camera (Photometrics) driven by Metamorph imaging software (Molecular Devices).

For time lapse video microscopy, cells were cotransfected with mCherry-actin and GFP-MRLC using Lipofectamine LTX supplemented with PLUS Reagent (Invitrogen). Transfected cells were plated onto 35 mm glass-bottomed dishes in phenol red–free L-15 medium (Gibco) supplemented with 10% FBS. Dishes were immobilized on the microscope stage to prevent movements during subsequent medium exchanges, and kept at 34°C during cell spreading and observation. An expressing cell was found after 1.5–2 hours of spreading, single images were acquired in DIC, red, and green channels and the medium was exchanged to one containing 100 µM blebbistatin. After 1 h incubation in dark, the blebbistatin-containing medium was replaced with normal medium after double wash, and time lapse imaging was started within 1 min after wash using Eclipse Ti inverted microscope (Nikon) with Plan Apo 100×1.4NA objective and Hamamatsu ORCA ER CCD camera (Hamamatsu Photonics, Japan) driven by NIS-Elements AR software (Laboratory Imaging, Czech Republic).

Quantification of protrusions was performed on phalloidin-stained cells. Lamellipodia and ruffles were identified by their peripheral localization and increased fluorescence relative to the adjacent lamella (∼ 20–30% increase for lamellipodia and >30% increase for ruffles). The lengths of lamellipodia or ruffle-containing segments of the cell perimeter were measured using ImageJ software and expressed as percentage of the cell perimeter for each individual cell). At least 30 cells were used per condition.

For quantification of the cytoskeleton-associated fraction of NMII, the total fluorescence intensity of NMII immunostaining in detergent-extracted cells was measured after background subtraction using Metamorph imaging software (Molecular Devices). The data were expressed as percentage of fluorescence intensity of control untreated cells from the same experiment. At least 25 cells were used per condition. The pp-MRLC fluorescence intensity profiles along the ∼9 nm lines drawn from the cell periphery toward the center were obtained from immunostained cells using the linescan tool in Metamorph. At least 30 linescans from 10 cells were used per condition. Microsoft Excel software was used for statistics and plot generation.

### Electron Microscopy

Samples for platinum replica EM, correlative light and EM, gelsolin treatment, and NMII immunogold staining were processed as described previously [49], [91], [92], [93]. Extraction solution contained 1% Triton X-100, 2% PEG (MW 35,000), 2 µM unlabeled phalloidin, and 2 µM paclitaxel in PEM buffer. For determination of cell surface topography, the extraction step was omitted. Secondary antibody conjugated with 12 nm or 18 nm colloidal gold (Jackson Immunoresearch Laboratories Inc.) were used for NMII immunostaining. For gelsolin treatment, detergent-extracted unfixed cells were incubated with 0.4 µg/ml gelsolin (gift of Dr. A. Weber, University of Pennsylvania). For immunogold NMII staining of gelsolin-treated samples, cells were incubated with the primary antibody in PEM buffer for 15 min after gelsolin treatment, rinsed in PEM and fixed.

Samples were analyzed using JEM 1011 transmission EM (JEOL USA, Peabody, MA) operated at 100 kV. In experiments involving non-correlative EM, dozens of cells per sample were analyzed to evaluate the generality and variability of a phenotype. For experiments with correlative EM, the conclusions were derived based on analysis of 5–10 cells. Images were captured by ORIUS 832.10W CCD camera (Gatan, Warrendale, PA) and presented in inverted contrast. Identification of gold particles in replica EM samples was performed at high magnification after contrast enhancement to distinguish them from other bright objects in the samples, such as actin filament tips. Thus, gold particles showed up as solid white circles in contrast to actin filament ends, which usually have a donut-like appearance. Overlaid images were prepared using Adobe Photoshop by placing the fluorescence image as a separate layer in a screen mode on the top of the replica EM image. Gold particles were highlighted using brush tool in Adobe Photoshop with 50% opacity. Color labeling of other structures of interest was performed using Hue/Saturation tool in Adobe Photoshop to avoid obscuring the structural details.

### Gradient Centrifugation and Western Blotting

Cells were grown in 60 mm Petri dishes overnight in a regular medium and either left untreated or were treated for 2 h with 100 µM blebbistatin. Then, cells were incubated for 10 min on ice with 200 µl of lysis buffer containing 20 mM HEPES (pH 6.9), 150 mM KCl, 1 mM DTT, 0.5% Triton-X100, and 1% Phosphatase Inhibitor Cocktail 3 (Sigma), and after incubation scraped off the dish into the same solution. The lysates were centrifuged at 68,000 rpm for 15 min in TLA100.2 rotor (Beckman) to remove detergent insoluble fraction. The total protein concentration in the supernatant was determined using Bradford method and equalized for control and blebbistatin-treated samples. The lysates were overlaid onto a discontinuous gradient of OptiPrep Density Gradient Medium (Sigma) in the lysis buffer consisting of 50%, 25%, 12%, and 6% layers. After centrifugation at 80,000 rpm for 60 min, 11 fractions (from top to bottom) were collected from each tube and mixed with the SDS-containing sample buffer (Invitrogen). All operations were carried out at 4°C. For Western blotting, proteins were separated by SDS-PAGE using 3–8% gradient gels (Invitrogen) for staining with the heavy chain NMII antibody or using 12% gel for staining with pp-MRLC antibody. After transfer to PVDF membranes, proteins were stained using appropriate primary antibodies and a secondary horseradish peroxidase-conjugated antibody and detected by ECL Prime Western Blotting Detection Reagents (Amersham). Quantification of the band density was performed by densitometry analysis.

## Supporting Information

Figure S1 **Effects of 75 µM blebbistatin on cell morphology.** (A) Cell surface topography revealed by platinum replica EM of non-extracted cell. Scale bar, 2 µm. (B–E) Fluorescence microscopy of phalloidin-stained F-actin and immunostained α-actinin (B), vinculin (C), NMII in pre-extracted cells (D) or in directly fixed cells (E). Scale bar, 20 µm. Boxed regions are zoomed in the bottom row.
(TIF)

Figure S2 (A) Effects of DMSO and (+)-blebbistatin on REF52 cells. Cell treatment with 1% DMSO or 100 µM of inactive (+)-blebbistatin does not affect organization of phalloidin-stained F-actin or immunostained NMII in REF52 cells. Scale bar, 20 µm. (B,C) Separation of NMII pools by gradient centrifugation followed by SDS-PAGE and Western blotting with NMII antibody. (B) Upper part shows Western blotting with NMII antibody of total cell lysates (TCL) of untreated (C) and (−)-blebbistatin-treated (BS) cells. Lower part sows representative Western blot of gradient fractions. (C) Average intensities of NMII bands in individual fractions after normalization to the total NMII in all fractions are plotted against the fraction number. Error bars, SD (N = 5 experiments). Cytosols of both untreated cells (pink) and cells treated with 100 µM active (−)-blebbistatin (blue) contain two subpopulations of NMII with peaks in fractions 4 and 8 with sedimentation coefficients corresponding to NMII monomers and NMII filaments, respectively. Relative distribution of soluble NMII between two peaks is similar in both conditions. Arrows indicate position of marker proteins: aldolase (7S); catalase (11 S) and ferritin (16S).
(TIF)

Figure S3 **Platinum replica EM of cells treated with 75 µM blebbistatin.** (A,B) Immunogold NMII staining (yellow dots) of cell periphery including lamellipodium and distal lamella (A) and of the proximal lamella (B). Microtubules are pseudocolored green. (C,D) EM of gelsolin-treated cytoskeleton without (C) or with (D) NMII immunogold labeling. Arrows indicate individual NMII filaments. Scale bars, 0.5 µm (A–C), 0.2 µm (D).
(TIF)

Figure S4 **Restoration of α-actinin organization after 100 µM blebbistatin washout.** Fluorescence microscopy of phalloidin-stained F-actin and immunostained α-actinin. Scale bar, 20 µm.
(TIF)

Figure S5 **Restoration of NMII organization after washout of 100 µM blebbistatin.** Fluorescence microscopy of phalloidin-stained F-actin and immunostained NMII in directly fixed cells. Boxed regions are zoomed in right column. Scale bar, 20 µm.
(TIF)

Figure S6 **Correlative fluorescence and EM of REF-52 cell recovering for 5 min after washout of 100 µM blebbistatin.** REF52 cell (the same as in Figure 11A–E) stained with phalloidin (not shown) and vinculin antibody (cyan) and additionally labeled with NMII immunogold. (A) Low magnification EM image overlaid with vinculin immunofluorescence in cyan. (B) Enlarged box from (A) showing multiple focal complexes (cyan spots) in lamella, some of which colocalize with concave arcs at the base of lamellipodia. (C,D) Enlarged boxes from B labeled by corresponding letters. Focal complexes in C may represent points of attachment of small actin filament bundles entering these regions. Boxed region enlarged in the inset shows accumulation of linear clusters of NMII immunogold particles in the associated bundle, indicating formation of NMII filaments. Focal complex in D resides at the junction of filopodial bundle with a concave arc. Bars, 10 µm (A); 2 µm (B); and 0.5 µm (C,D).
(TIF)

Figure S7 **Actin polymerization is required for efficient restoration of the contractile system.** Fluorescence microscopy of phalloidin-stained F-actin and immunostained vinculin in blebbistatin or/and latrunculin treated cells (A) and cells after blebbistatin washout in presence of latrunculin (B). Scale bar, 20 µm.
(TIF)

Video S1 Time lapse video of a cell cotransfected with mCherry-actin and GFP-MRLC treated with 100 µM blebbistatin and washed out of the drug.
(MOV)

## References

[1] Gautel M (2011). The sarcomeric cytoskeleton: who picks up the strain?. Curr Opin Cell Biol.

[2] Parsons JT, Horwitz AR, Schwartz MA (2010). Cell adhesion: integrating cytoskeletal dynamics and cellular tension.. Nat Rev Mol Cell Biol.

[3] Vicente-Manzanares M, Ma X, Adelstein RS, Horwitz AR (2009). Non-muscle myosin II takes centre stage in cell adhesion and migration.. Nat Rev Mol Cell Biol.

[4] Wolfenson H, Henis YI, Geiger B, Bershadsky AD (2009). The heel and toe of the cell's foot: a multifaceted approach for understanding the structure and dynamics of focal adhesions.. Cell Motil Cytoskeleton.

[5] Sanger JW, Wang J, Fan Y, White J, Sanger JM (2010). Assembly and dynamics of myofibrils.. Journal of biomedicine & biotechnology.

[6] Matsumura F (2005). Regulation of myosin II during cytokinesis in higher eukaryotes.. Trends Cell Biol.

[7] Ikebe M, Hartshorne DJ, Elzinga M (1986). Identification, phosphorylation, and dephosphorylation of a second site for myosin light chain kinase on the 20,000-dalton light chain of smooth muscle myosin.. J Biol Chem.

[8] Clark K, Langeslag M, Figdor CG, van Leeuwen FN (2007). Myosin II and mechanotransduction: a balancing act.. Trends Cell Biol.

[9] Pollard TD, Borisy GG (2003). Cellular motility driven by assembly and disassembly of actin filaments.. Cell.

[10] Choi CK, Vicente-Manzanares M, Zareno J, Whitmore LA, Mogilner A (2008). Actin and alpha-actinin orchestrate the assembly and maturation of nascent adhesions in a myosin II motor-independent manner.. Nat Cell Biol.

[11] Rottner K, Hall A, Small JV (1999). Interplay between Rac and Rho in the control of substrate contact dynamics.. Curr Biol.

[12] Bershadsky A, Kozlov M, Geiger B (2006). Adhesion-mediated mechanosensitivity: a time to experiment, and a time to theorize.. Curr Opin Cell Biol.

[13] Chrzanowska-Wodnicka M, Burridge K (1996). Rho-stimulated contractility drives the formation of stress fibers and focal adhesions.. J Cell Biol.

[14] Riveline D, Zamir E, Balaban NQ, Schwarz US, Ishizaki T (2001). Focal contacts as mechanosensors: externally applied local mechanical force induces growth of focal contacts by an mDia1-dependent and ROCK-independent mechanism.. J Cell Biol.

[15] Alexandrova AY, Arnold K, Schaub S, Vasiliev JM, Meister JJ (2008). Comparative dynamics of retrograde actin flow and focal adhesions: formation of nascent adhesions triggers transition from fast to slow flow.. PLoS One.

[16] Gardel ML, Sabass B, Ji L, Danuser G, Schwarz US (2008). Traction stress in focal adhesions correlates biphasically with actin retrograde flow speed.. J Cell Biol.

[17] Svitkina TM, Verkhovsky AB, McQuade KM, Borisy GG (1997). Analysis of the actin-myosin II system in fish epidermal keratocytes: mechanism of cell body translocation.. J Cell Biol.

[18] Verkhovsky AB, Svitkina TM, Borisy GG (1995). Myosin II filament assemblies in the active lamella of fibroblasts: their morphogenesis and role in the formation of actin filament bundles.. J Cell Biol.

[19] Anderson TW, Vaughan AN, Cramer LP (2008). Retrograde flow and myosin II activity within the leading cell edge deliver F-actin to the lamella to seed the formation of graded polarity actomyosin II filament bundles in migrating fibroblasts.. Mol Biol Cell.

[20] Nemethova M, Auinger S, Small JV (2008). Building the actin cytoskeleton: filopodia contribute to the construction of contractile bundles in the lamella.. J Cell Biol.

[21] Hotulainen P, Lappalainen P (2006). Stress fibers are generated by two distinct actin assembly mechanisms in motile cells.. J Cell Biol.

[22] Tojkander S, Gateva G, Schevzov G, Hotulainen P, Naumanen P (2011). A molecular pathway for myosin II recruitment to stress fibers.. Curr Biol.

[23] Straight AF, Cheung A, Limouze J, Chen I, Westwood NJ (2003). Dissecting temporal and spatial control of cytokinesis with a myosin II Inhibitor.. Science.

[24] Kovacs M, Toth J, Hetenyi C, Malnasi-Csizmadia A, Sellers JR (2004). Mechanism of blebbistatin inhibition of myosin II.. J Biol Chem.

[25] Verkhovsky AB, Svitkina TM, Borisy GG (1997). Polarity sorting of actin filaments in cytochalasin-treated fibroblasts.. J Cell Sci 110 (Pt.

[26] Betapudi V, Licate LS, Egelhoff TT (2006). Distinct roles of nonmuscle myosin II isoforms in the regulation of MDA-MB-231 breast cancer cell spreading and migration.. Cancer Res.

[27] Iwasaki T, Wang YL (2008). Cytoplasmic force gradient in migrating adhesive cells.. Biophys J.

[28] Koestler SA, Auinger S, Vinzenz M, Rottner K, Small JV (2008). Differentially oriented populations of actin filaments generated in lamellipodia collaborate in pushing and pausing at the cell front.. Nat Cell Biol.

[29] Kolega J (2006). The role of myosin II motor activity in distributing myosin asymmetrically and coupling protrusive activity to cell translocation.. Mol Biol Cell.

[30] Kondo T, Hamao K, Kamijo K, Kimura H, Morita M (2011). Enhancement of myosin II/actin turnover at the contractile ring induces slower furrowing in dividing HeLa cells.. Biochem J.

[31] Sandquist JC, Means AR (2008). The C-terminal tail region of nonmuscle myosin II directs isoform-specific distribution in migrating cells.. Mol Biol Cell.

[32] Schiller HB, Friedel CC, Boulegue C, Fassler R (2011). Quantitative proteomics of the integrin adhesome show a myosin II-dependent recruitment of LIM domain proteins.. EMBO Rep.

[33] Watanabe T, Hosoya H, Yonemura S (2007). Regulation of myosin II dynamics by phosphorylation and dephosphorylation of its light chain in epithelial cells.. Mol Biol Cell.

[34] Shu S, Liu X, Korn ED (2005). Blebbistatin and blebbistatin-inactivated myosin II inhibit myosin II-independent processes in Dictyostelium.. Proc Natl Acad Sci U S A.

[35] Shutova MS, Alexandrova AY, Vasiliev JM (2008). Regulation of polarity in cells devoid of actin bundle system after treatment with inhibitors of myosin II activity.. Cell Motil Cytoskeleton.

[36] Otey CA, Carpen O (2004). Alpha-actinin revisited: a fresh look at an old player.. Cell Motil Cytoskeleton.

[37] Zaidel-Bar R, Geiger B (2010). The switchable integrin adhesome.. J Cell Sci.

[38] Cai Y, Rossier O, Gauthier NC, Biais N, Fardin MA (2010). Cytoskeletal coherence requires myosin-IIA contractility.. J Cell Sci.

[39] Pasapera AM, Schneider IC, Rericha E, Schlaepfer DD, Waterman CM (2010). Myosin II activity regulates vinculin recruitment to focal adhesions through FAK-mediated paxillin phosphorylation.. J Cell Biol.

[40] Sandquist JC, Swenson KI, Demali KA, Burridge K, Means AR (2006). Rho kinase differentially regulates phosphorylation of nonmuscle myosin II isoforms A and B during cell rounding and migration.. J Biol Chem.

[41] Totsukawa G, Wu Y, Sasaki Y, Hartshorne DJ, Yamakita Y (2004). Distinct roles of MLCK and ROCK in the regulation of membrane protrusions and focal adhesion dynamics during cell migration of fibroblasts.. J Cell Biol.

[42] Craig R, Smith R, Kendrick-Jones J (1983). Light-chain phosphorylation controls the conformation of vertebrate non-muscle and smooth muscle myosin molecules.. Nature.

[43] Sinard JH, Stafford WF, Pollard TD (1989). The mechanism of assembly of Acanthamoeba myosin-II minifilaments: minifilaments assemble by three successive dimerization steps.. J Cell Biol.

[44] Hale CM, Sun SX, Wirtz D (2009). Resolving the role of actomyosin contractility in cell microrheology.. PLoS One.

[45] Watanabe M, Yumoto M, Tanaka H, Wang HH, Katayama T (2010). Blebbistatin, a myosin II inhibitor, suppresses contraction and disrupts contractile filaments organization of skinned taenia cecum from guinea pig.. Am J Physiol Cell Physiol.

[46] Svitkina TM, Borisy GG (1999). Arp2/3 complex and actin depolymerizing factor/cofilin in dendritic organization and treadmilling of actin filament array in lamellipodia.. J Cell Biol.

[47] Svitkina TM, Shevelev AA, Bershadsky AD, Gelfand VI (1984). Cytoskeleton of mouse embryo fibroblasts. Electron microscopy of platinum replicas.. Eur J Cell Biol.

[48] Svitkina TM, Surguchova IG, Verkhovsky AB, Gelfand VI, Moeremans M (1989). Direct visualization of bipolar myosin filaments in stress fibers of cultured fibroblasts.. Cell Motil Cytoskeleton.

[49] Svitkina TM, Borisy GG (1998). Correlative light and electron microscopy of the cytoskeleton of cultured cells.. Methods Enzymol.

[50] Shemesh T, Verkhovsky AB, Svitkina TM, Bershadsky AD, Kozlov MM (2009). Role of focal adhesions and mechanical stresses in the formation and progression of the lamellipodium-lamellum interface [corrected].. Biophys J.

[51] Pizon V, Gerbal F, Diaz CC, Karsenti E (2005). Microtubule-dependent transport and organization of sarcomeric myosin during skeletal muscle differentiation.. Embo J.

[52] Kolega J (2004). Phototoxicity and photoinactivation of blebbistatin in UV and visible light.. Biochem Biophys Res Commun.

[53] Sakamoto T, Limouze J, Combs CA, Straight AF, Sellers JR (2005). Blebbistatin, a myosin II inhibitor, is photoinactivated by blue light.. Biochemistry.

[54] Yu HY, Bement WM (2007). Multiple myosins are required to coordinate actin assembly with coat compression during compensatory endocytosis.. Mol Biol Cell.

[55] Takacs B, Billington N, Gyimesi M, Kintses B, Malnasi-Csizmadia A (2010). Myosin complexed with ADP and blebbistatin reversibly adopts a conformation resembling the start point of the working stroke.. Proc Natl Acad Sci U S A.

[56] Kovacs M, Thirumurugan K, Knight PJ, Sellers JR (2007). Load-dependent mechanism of nonmuscle myosin 2.. Proc Natl Acad Sci U S A.

[57] Bridgman PC, Dave S, Asnes CF, Tullio AN, Adelstein RS (2001). Myosin IIB is required for growth cone motility.. J Neurosci.

[58] Li ZH, Bresnick AR (2006). The S100A4 metastasis factor regulates cellular motility via a direct interaction with myosin-IIA.. Cancer Res.

[59] Morimura S, Suzuki K, Takahashi K (2011). Nonmuscle myosin IIA is required for lamellipodia formation through binding to WAVE2 and phosphatidylinositol 3,4,5-triphosphate.. Biochem Biophys Res Commun.

[60] Even-Ram S, Doyle AD, Conti MA, Matsumoto K, Adelstein RS (2007). Myosin IIA regulates cell motility and actomyosin-microtubule crosstalk.. Nat Cell Biol.

[61] Lee CS, Choi CK, Shin EY, Schwartz MA, Kim EG (2010). Myosin II directly binds and inhibits Dbl family guanine nucleotide exchange factors: a possible link to Rho family GTPases.. J Cell Biol.

[62] Jurado C, Haserick JR, Lee J (2005). Slipping or gripping? Fluorescent speckle microscopy in fish keratocytes reveals two different mechanisms for generating a retrograde flow of actin.. Mol Biol Cell.

[63] Tsuji T, Ishizaki T, Okamoto M, Higashida C, Kimura K (2002). ROCK and mDia1 antagonize in Rho-dependent Rac activation in Swiss 3T3 fibroblasts.. J Cell Biol.

[64] Vicente-Manzanares M, Zareno J, Whitmore L, Choi CK, Horwitz AF (2007). Regulation of protrusion, adhesion dynamics, and polarity by myosins IIA and IIB in migrating cells.. J Cell Biol.

[65] Conti MA, Even-Ram S, Liu C, Yamada KM, Adelstein RS (2004). Defects in cell adhesion and the visceral endoderm following ablation of nonmuscle myosin heavy chain II-A in mice.. J Biol Chem.

[66] Ma X, Jana SS, Conti MA, Kawamoto S, Claycomb WC (2010). Ablation of nonmuscle myosin II-B and II-C reveals a role for nonmuscle myosin II in cardiac myocyte karyokinesis.. Mol Biol Cell.

[67] Beningo KA, Dembo M, Kaverina I, Small JV, Wang YL (2001). Nascent focal adhesions are responsible for the generation of strong propulsive forces in migrating fibroblasts.. J Cell Biol.

[68] Henson JH, Svitkina TM, Burns AR, Hughes HE, MacPartland KJ (1999). Two components of actin-based retrograde flow in sea urchin coelomocytes.. Mol Biol Cell.

[69] Medeiros NA, Burnette DT, Forscher P (2006). Myosin II functions in actin-bundle turnover in neuronal growth cones.. Nat Cell Biol.

[70] Schaub S, Bohnet S, Laurent VM, Meister JJ, Verkhovsky AB (2007). Comparative maps of motion and assembly of filamentous actin and myosin II in migrating cells.. Mol Biol Cell.

[71] Wilson CA, Tsuchida MA, Allen GM, Barnhart EL, Applegate KT (2010). Myosin II contributes to cell-scale actin network treadmilling through network disassembly.. Nature.

[72] Kim EJ, Helfman DM (2003). Characterization of the metastasis-associated protein, S100A4. Roles of calcium binding and dimerization in cellular localization and interaction with myosin.. J Biol Chem.

[73] Matsumura F, Ono S, Yamakita Y, Totsukawa G, Yamashiro S (1998). Specific localization of serine 19 phosphorylated myosin II during cell locomotion and mitosis of cultured cells.. J Cell Biol.

[74] Dahan I, Yearim A, Touboul Y, Ravid S (2012). The tumor suppressor Lgl1 regulates NMII-A cellular distribution and focal adhesion morphology to optimize cell migration.. Mol Biol Cell.

[75] Rosenberg M, Ravid S (2006). Protein kinase Cgamma regulates myosin IIB phosphorylation, cellular localization, and filament assembly.. Mol Biol Cell.

[76] Sanborn KB, Mace EM, Rak GD, Difeo A, Martignetti JA (2011). Phosphorylation of the myosin IIA tailpiece regulates single myosin IIA molecule association with lytic granules to promote NK-cell cytotoxicity.. Blood.

[77] Li D, Miller M, Chantler PD (1994). Association of a cellular myosin II with anionic phospholipids and the neuronal plasma membrane.. Proc Natl Acad Sci U S A.

[78] Rosado LA, Horn TA, McGrath SC, Cotter RJ, Yang JT (2011). Association between {alpha}4 integrin cytoplasmic tail and non-muscle myosin IIA regulates cell migration.. J Cell Sci.

[79] Sun SX, Walcott S, Wolgemuth CW (2010). Cytoskeletal cross-linking and bundling in motor-independent contraction.. Curr Biol.

[80] Luo T, Mohan K, Srivastava V, Ren Y, Iglesias PA (2012). Understanding the cooperative interaction between myosin II and actin cross-linkers mediated by actin filaments during mechanosensation.. Biophys J.

[81] Ren Y, Effler JC, Norstrom M, Luo T, Firtel RA (2009). Mechanosensing through cooperative interactions between myosin II and the actin crosslinker cortexillin I. Curr Biol.

[82] Fernandez-Gonzalez R, Simoes Sde M, Roper JC, Eaton S, Zallen JA (2009). Myosin II dynamics are regulated by tension in intercalating cells.. Dev Cell.

[83] Goeckeler ZM, Bridgman PC, Wysolmerski RB (2008). Nonmuscle myosin II is responsible for maintaining endothelial cell basal tone and stress fiber integrity.. Am J Physiol Cell Physiol.

[84] Wu C, Asokan SB, Berginski ME, Haynes EM, Sharpless NE (2012). Arp2/3 is critical for lamellipodia and response to extracellular matrix cues but is dispensable for chemotaxis.. Cell.

[85] Uyeda TQ, Iwadate Y, Umeki N, Nagasaki A, Yumura S (2011). Stretching actin filaments within cells enhances their affinity for the myosin II motor domain.. PloS One.

[86] Heath JP (1981). Arcs: curved microfilament bundles beneath the dorsal surface of the leading lamellae of moving chick embryo fibroblasts.. Cell Biol Int Rep.

[87] Aratyn-Schaus Y, Oakes PW, Gardel ML (2011). Dynamic and structural signatures of lamellar actomyosin force generation.. Mol Biol Cell.

[88] Verkhovsky AB, Surgucheva IG, Svitkina TM, Tint IS, Gelfand VI (1987). Organization of stress fibers in cultured fibroblasts after extraction of actin with bovine brain gelsolin-like protein.. Exp Cell Res.

[89] Zeng L, Xu H, Chew TL, Eng E, Sadeghi MM (2005). HMG CoA reductase inhibition modulates VEGF-induced endothelial cell hyperpermeability by preventing RhoA activation and myosin regulatory light chain phosphorylation.. FASEB J.

[90] Yang C, Hoelzle M, Disanza A, Scita G, Svitkina T (2009). Coordination of membrane and actin cytoskeleton dynamics during filopodia protrusion.. PLoS One.

[91] Svitkina TM, Borisy GG, Celis J (2006). Correlative light and electron microscopy studies of cytoskeletal dynamics..

[92] Svitkina T (2007). Electron microscopic analysis of the leading edge in migrating cells.. Methods Cell Biol.

[93] Svitkina T (2009). Imaging cytoskeleton components by electron microscopy.. Methods Mol Biol.

