# Supplementary figures and images for: Functions of Nonmuscle Myosin II in Assembly of the Cellular Contractile System
Source: PLoS One. 2012 Jul 13;7(7):e40814. doi: 10.1371/journal.pone.0040814 (PMC3396643; doi:10.1371/journal.pone.0040814)

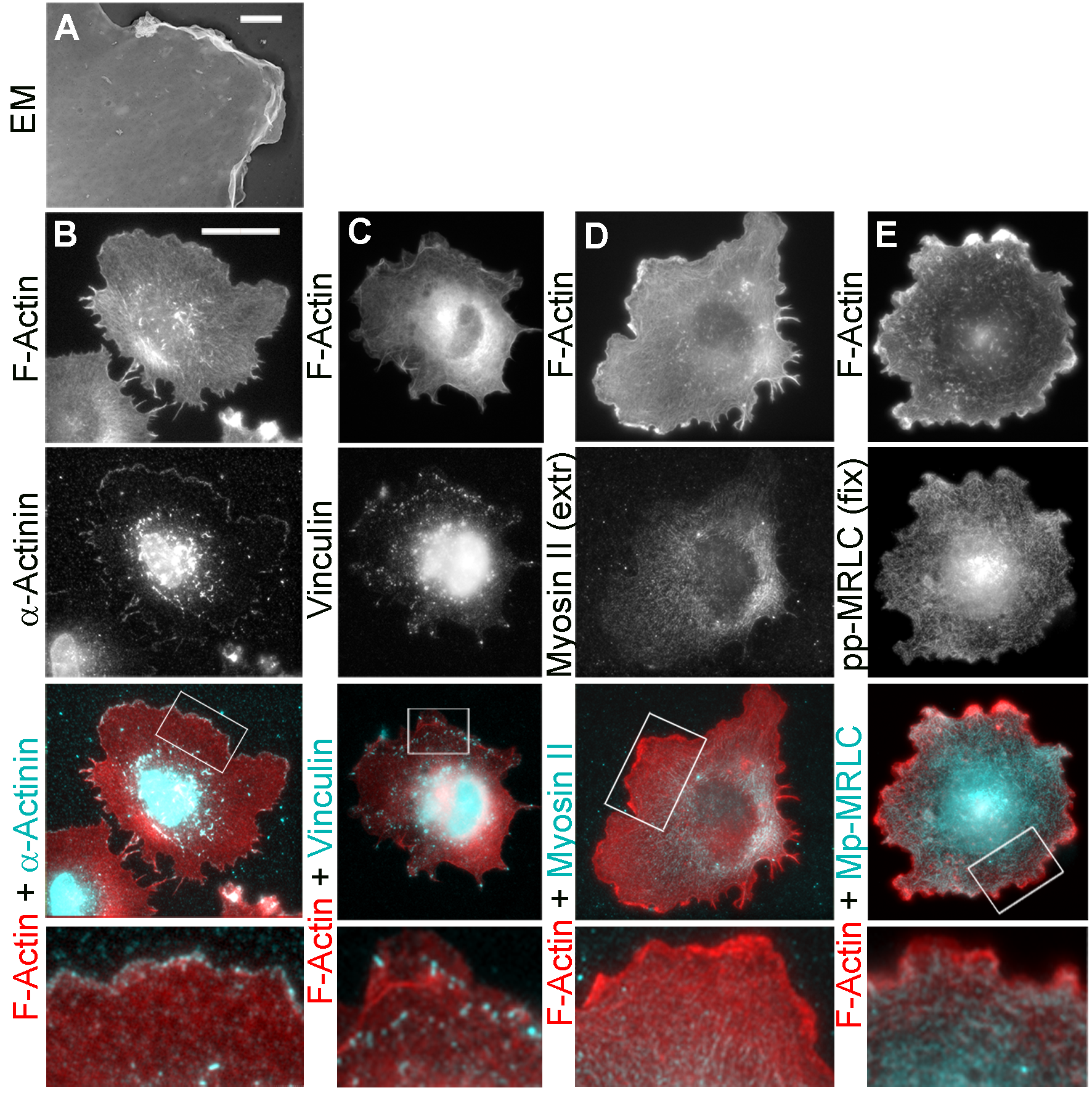

Supplement: Figure S1 — Effects of 75 µM blebbistatin on cell morphology. (A) Cell surface topography revealed by platinum replica EM of non-extracted cell. Scale bar, 2 µm. (B–E) Fluorescence microscopy of phalloidin-stained F-actin and immunostained α-actinin (B), vinculin (C), NMII in pre-extracted cells (D) or in directly fixed cells (E). Scale bar, 20 µm. Boxed regions are zoomed in the bottom row. (TIF) [file pone.0040814.s001.tif]

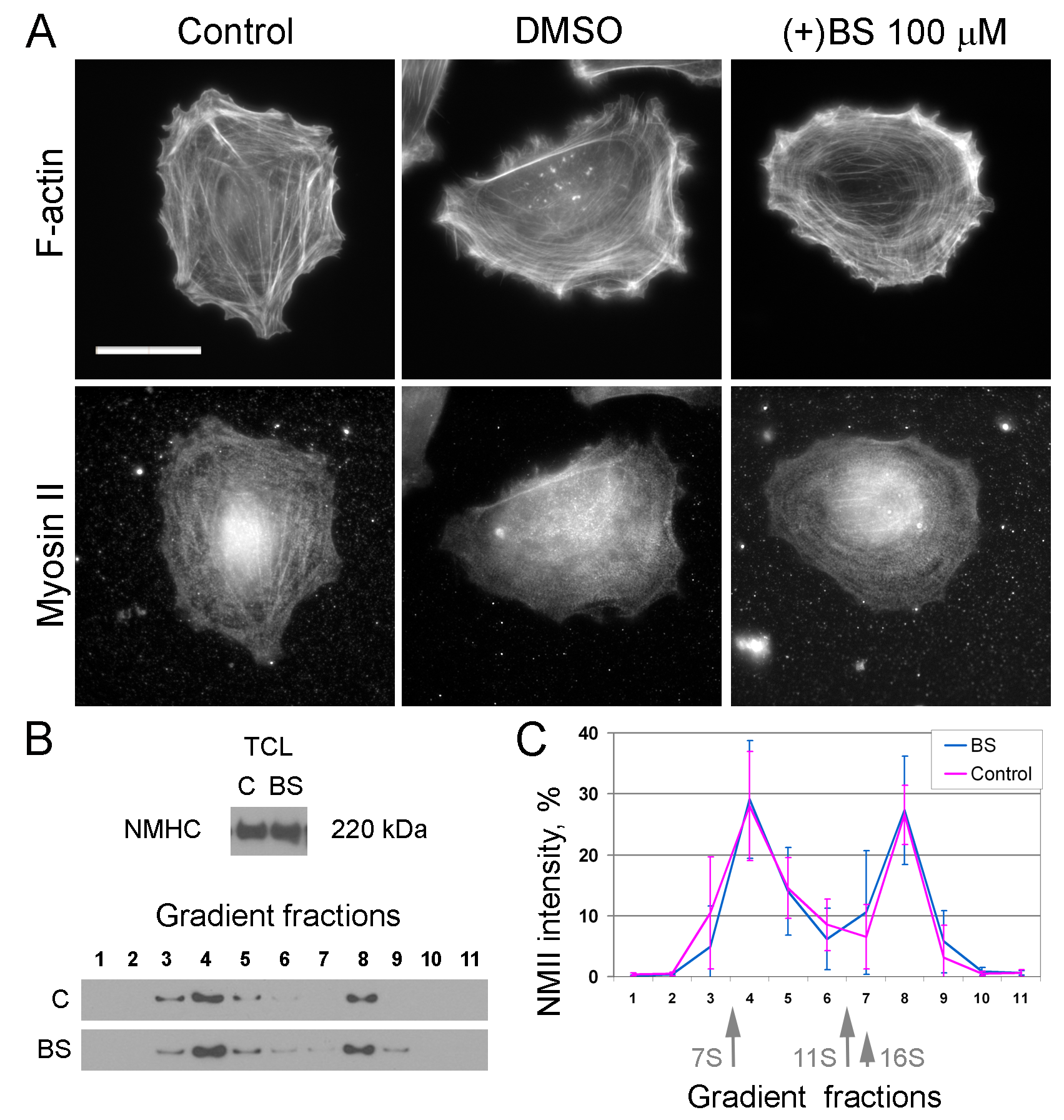

Supplement: Figure S2 — (A) Effects of DMSO and (+)-blebbistatin on REF52 cells. Cell treatment with 1% DMSO or 100 µM of inactive (+)-blebbistatin does not affect organization of phalloidin-stained F-actin or immunostained NMII in REF52 cells. Scale bar, 20 µm. (B,C) Separation of NMII pools by gradient centrifugation followed by SDS-PAGE and Western blotting with NMII antibody. (B) Upper part shows Western blotting with NMII antibody of total cell lysates (TCL) of untreated (C) and (−)-blebbistatin-treated (BS) cells. Lower part sows representative Western blot of gradient fractions. (C) Average intensities of NMII bands in individual fractions after normalization to the total NMII in all fractions are plotted against the fraction number. Error bars, SD (N = 5 experiments). Cytosols of both untreated cells (pink) and cells treated with 100 µM active (−)-blebbistatin (blue) contain two subpopulations of NMII with peaks in fractions 4 and 8 with sedimentation coefficients corresponding to NMII monomers and NMII filaments, respectively. Relative distribution of soluble NMII between two peaks is similar in both conditions. Arrows indicate position of marker proteins: aldolase (7S); catalase (11 S) and ferritin (16S). (TIF) [file pone.0040814.s002.tif]

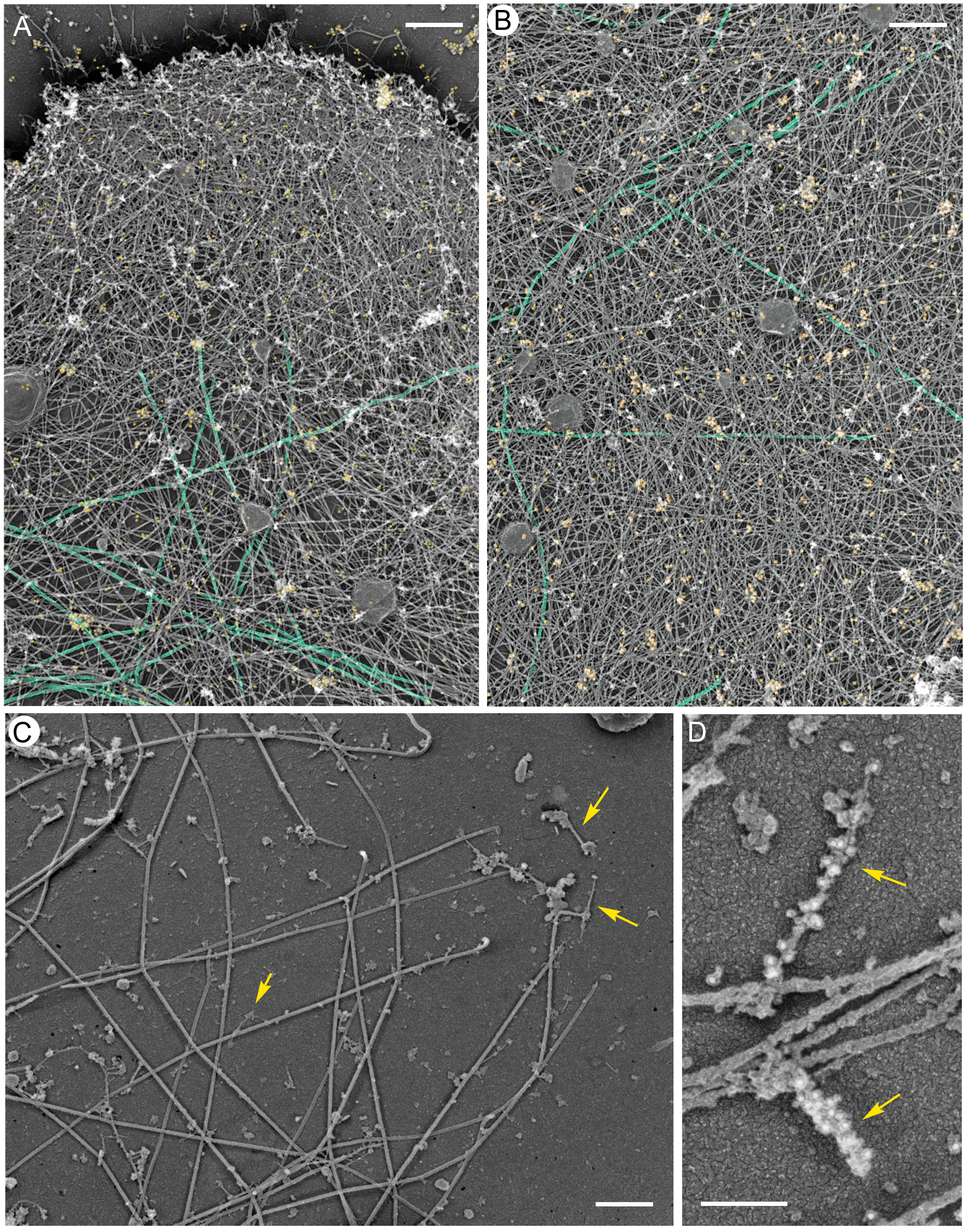

Supplement: Figure S3 — Platinum replica EM of cells treated with 75 µM blebbistatin. (A,B) Immunogold NMII staining (yellow dots) of cell periphery including lamellipodium and distal lamella (A) and of the proximal lamella (B). Microtubules are pseudocolored green. (C,D) EM of gelsolin-treated cytoskeleton without (C) or with (D) NMII immunogold labeling. Arrows indicate individual NMII filaments. Scale bars, 0.5 µm (A–C), 0.2 µm (D). (TIF) [file pone.0040814.s003.tif]

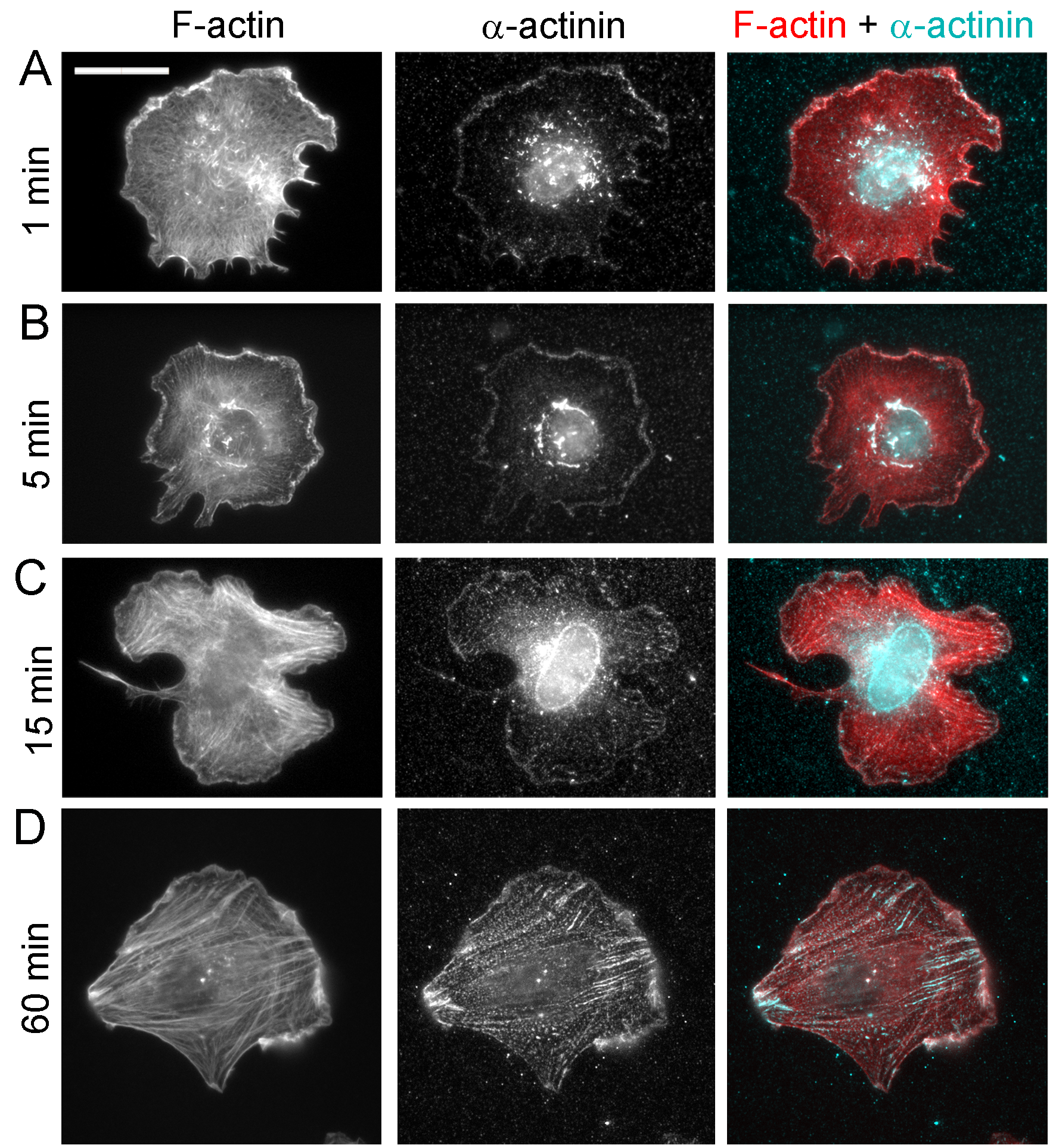

Supplement: Figure S4 — Restoration of α-actinin organization after 100 µM blebbistatin washout. Fluorescence microscopy of phalloidin-stained F-actin and immunostained α-actinin. Scale bar, 20 µm. (TIF) [file pone.0040814.s004.tif]

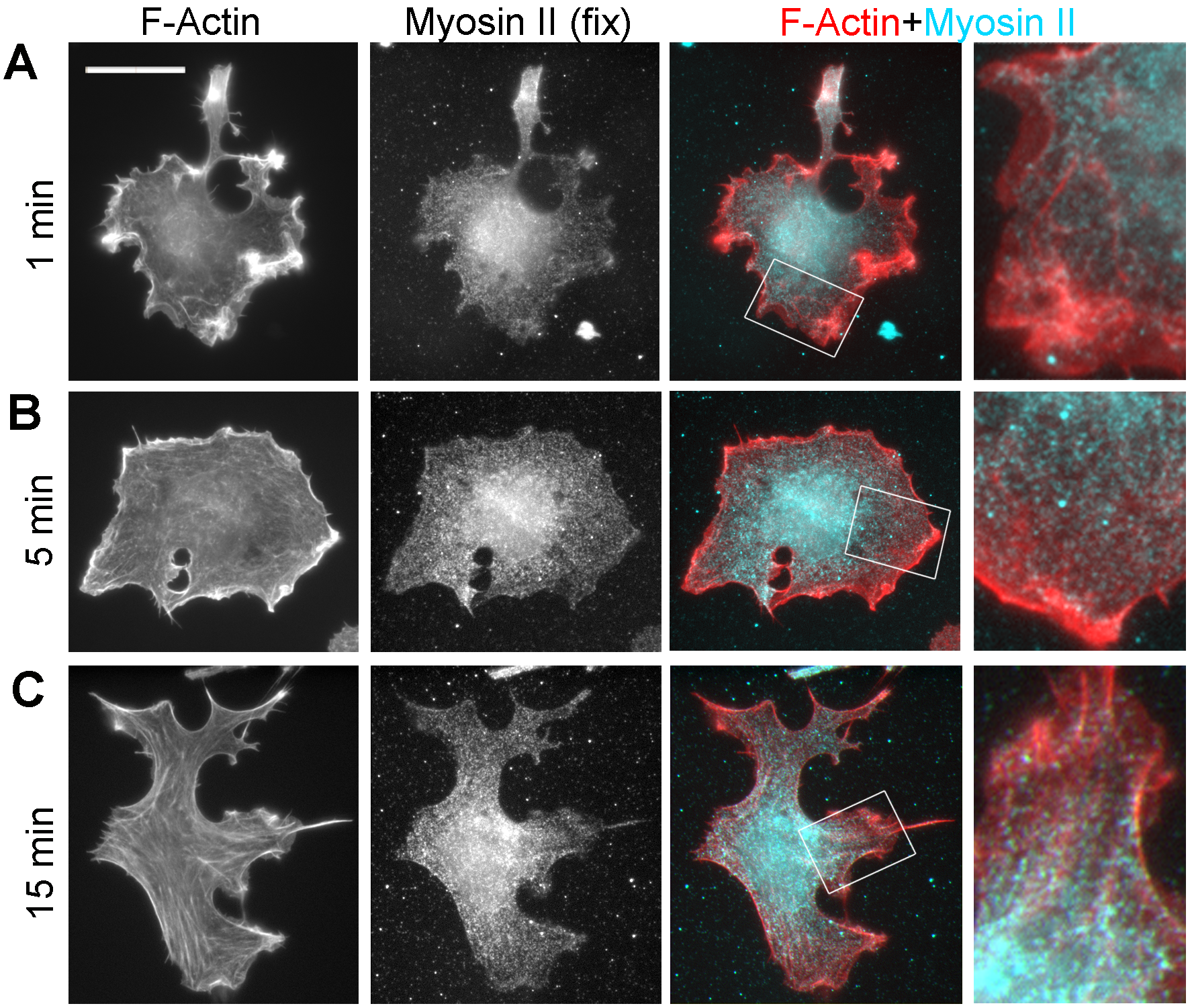

Supplement: Figure S5 — Restoration of NMII organization after washout of 100 µM blebbistatin. Fluorescence microscopy of phalloidin-stained F-actin and immunostained NMII in directly fixed cells. Boxed regions are zoomed in right column. Scale bar, 20 µm. (TIF) [file pone.0040814.s005.tif]

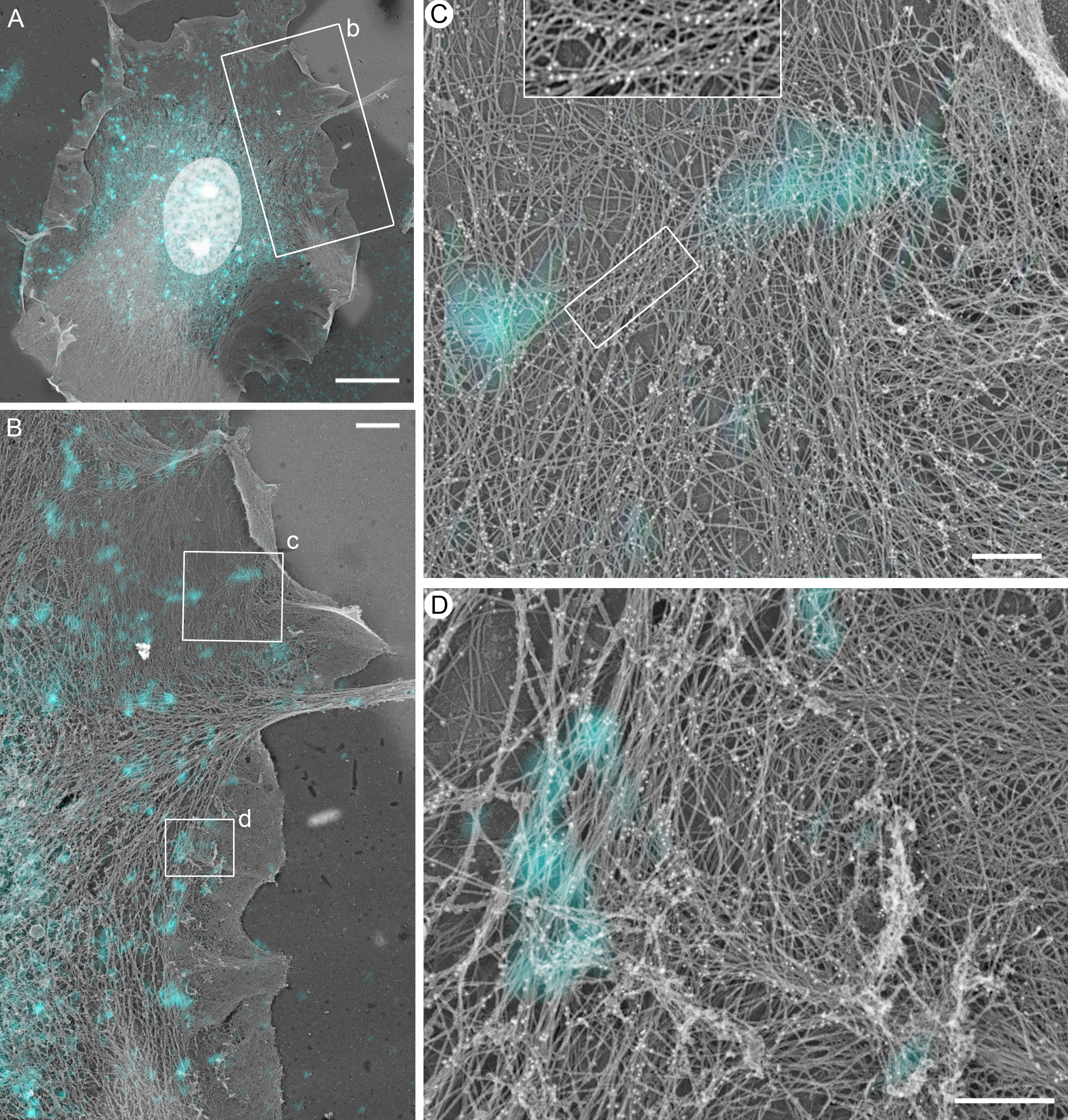

Supplement: Figure S6 — Correlative fluorescence and EM of REF-52 cell recovering for 5 min after washout of 100 µM blebbistatin. REF52 cell (the same as in Figure 11A–E) stained with phalloidin (not shown) and vinculin antibody (cyan) and additionally labeled with NMII immunogold. (A) Low magnification EM image overlaid with vinculin immunofluorescence in cyan. (B) Enlarged box from (A) showing multiple focal complexes (cyan spots) in lamella, some of which colocalize with concave arcs at the base of lamellipodia. (C,D) Enlarged boxes from B labeled by corresponding letters. Focal complexes in C may represent points of attachment of small actin filament bundles entering these regions. Boxed region enlarged in the inset shows accumulation of linear clusters of NMII immunogold particles in the associated bundle, indicating formation of NMII filaments. Focal complex in D resides at the junction of filopodial bundle with a concave arc. Bars, 10 µm (A); 2 µm (B); and 0.5 µm (C,D). (TIF) [file pone.0040814.s006.tif]

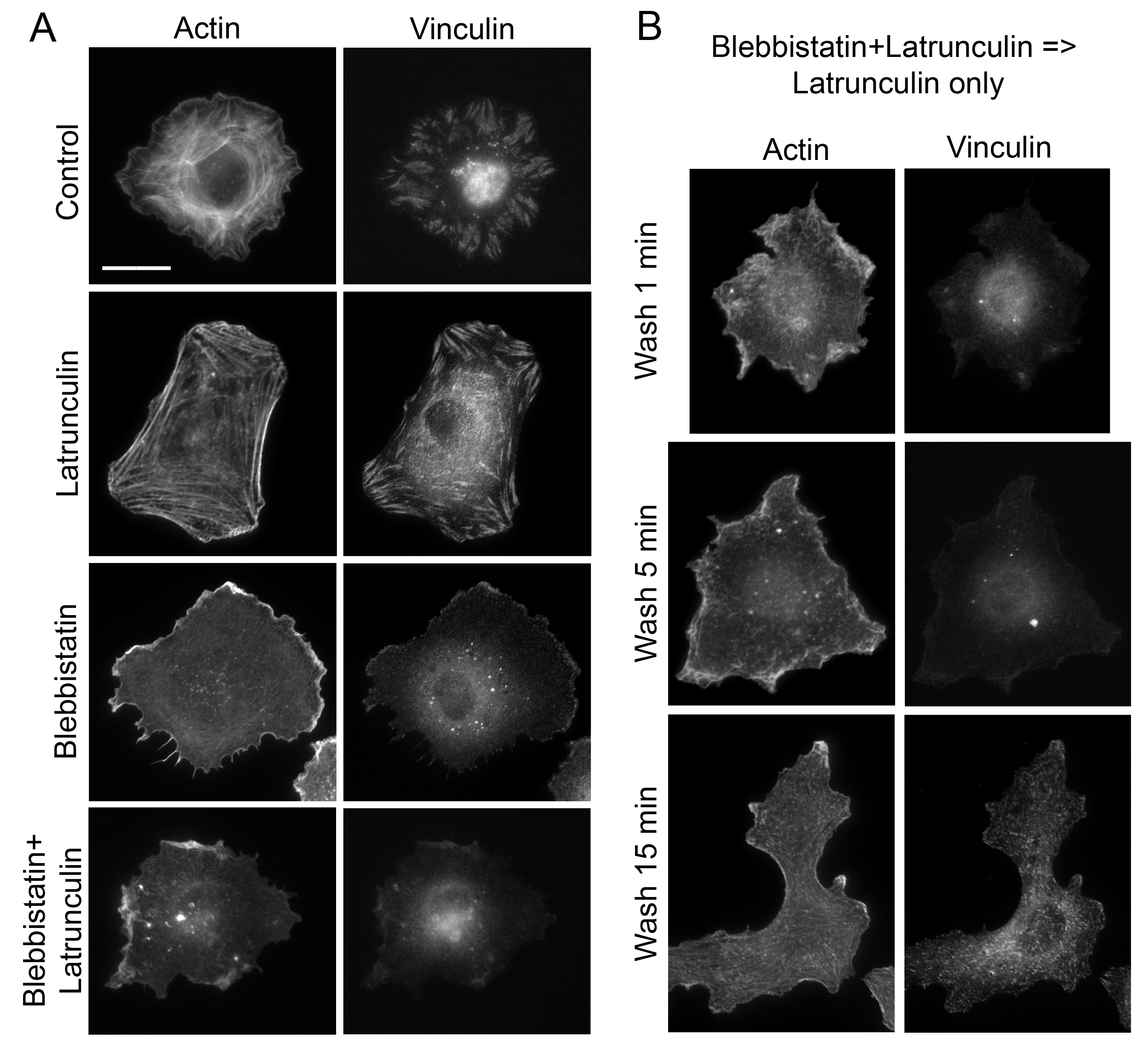

Supplement: Figure S7 — Actin polymerization is required for efficient restoration of the contractile system. Fluorescence microscopy of phalloidin-stained F-actin and immunostained vinculin in blebbistatin or/and latrunculin treated cells (A) and cells after blebbistatin washout in presence of latrunculin (B). Scale bar, 20 µm. (TIF) [file pone.0040814.s007.tif]
